# RUNX1 restrains STAT1-GITRL signaling to shape an immunosuppressive CRC microenvironment

**DOI:** 10.1038/s41420-026-03053-7

**Published:** 2026-03-25

**Authors:** Wenting He, Lisheng Zheng, Weiye Huang, Keren Li, Yitian Chen, Rui Zeng, Zhihao Lin, Yangwei Xu, Shengfeng Hu, Qingling Zhang

**Affiliations:** 1 Guangdong Cardiovascular Institute, Guangzhou, China; 2 Department of Pathology, Guangdong Provincial People’s Hospital, Guangdong Academy of Medical Sciences, Guangzhou, China; 3 Hepatopancereatobiliary Center, Tsinghua University Affiliated Beijing Tsinghua Changgung Hospital, School of Clinical Medicine, Tsinghua University, Beijing, China; 4 The Second Affiliated Hospital, The State Key Laboratory of Respiratory Disease, Guangdong Provincial Key Laboratory of Allergy & Clinical Immunology, Guangzhou Medical University, Guangzhou, China

**Keywords:** Colorectal cancer, Immunosurveillance

## Abstract

The oncogenic role of RUNX1 in epithelial tumors is increasingly recognized, however, its function and mechanism within the tumor immune microenvironment (TME) of colorectal cancer (CRC) remain unclear. This study investigates the contribution of RUNX1 to TME remodeling in CRC. Analysis of clinical CRC tissues revealed that RUNX1 expression is negatively correlated with GITRL levels in tumor cells and is associated with increased infiltration of Treg cells. Functional studies demonstrated that RUNX1 impairs GITRL-GITR signaling, thereby promoting Treg cell infiltration while suppressing CD8^+^ T cell activation. Consequently, elevated RUNX1 expression enhanced the sensitivity of CRC tumors to GITR agonistic antibody therapy in a C57BL/6J mouse model. Mechanistically, RUNX1 interacts with STAT1 to inhibit its dimerization and subsequent transcriptional activation of GITRL, thereby suppressing GITRL expression. Our findings highlight the RUNX1/STAT1/GITRL axis as a potential therapeutic target for GITR-based immunotherapy in CRC.

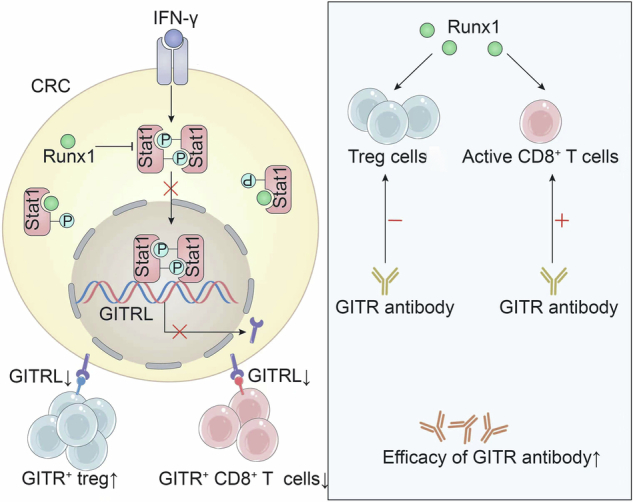

## Introduction

Colorectal cancer (CRC) remains a leading cause of global cancer-related mortality. Approximately 25% of patients are diagnosed at an advanced stage, exhibiting a median five‑year survival rate as low as 12.5%. Moreover, 25–50% of individuals initially diagnosed with early‑stage disease eventually experience recurrence [1–3]. Over the past decade, immune checkpoint blockade (ICB) therapies, particularly those targeting Programmed Death Receptor 1 (PD‑1), have achieved durable responses in various refractory solid tumors. In 2017, PD‑1 inhibitors gained U.S. Food and Drug Administration (FDA) approval for the treatment of CRC with high microsatellite instability (MSI‑H). However, approximately 50% of MSI‑H and 80% of microsatellite stable (MSS) or MSI‑low (MSI‑L) CRC patients fail to benefit from PD‑1‑based immunotherapy [4]. This suboptimal response is attributed to factors such as inadequate immune cell infiltration, diminished expression of inhibitory ligands, local immunosuppression, and pronounced exhaustion of cytotoxic T lymphocytes (CTLs) [5–7]. Therefore, there is an urgent need to develop effective immunotherapeutic strategies capable of remodeling the compromised tumor immune microenvironment (TME) in CRC patients.

Enhancing anti-tumor immunity through the activation of co-stimulatory receptors (CoRs) on tumor-infiltrating lymphocytes (TILs) represents a promising strategy that may complement existing ICB therapies. Beyond T-cell receptor (TCR) signaling, co-stimulatory signals are critical for driving robust T-cell activation, polarization into functional subsets, effector function, and long-term survival [8]. Among CoRs, several members of the tumor necrosis factor receptor (TNFR) superfamily (TNFRSF)—including CD30, DR3, GITR, HVEM, OX-40, TNFR3, and 4-1BB—have been extensively investigated for their potential in cancer immunotherapy [9, 10]. In particular, glucocorticoid-induced TNF receptor-related protein (GITR) has emerged as a key immunomodulatory checkpoint within the TNFRSF. GITR engagement has been shown to promote effector T-cell activity, inhibit regulatory T-cell (Treg) suppression, and induce potent anti-tumor responses [11–15].

The Runt-related transcription factor 1 (RUNX1) is a member of the evolutionarily conserved RUNX family of transcriptional regulators, which play pivotal roles in cell fate determination and tissue development across diverse lineages [16–18]. In hematological malignancies, RUNX1 is among the most frequently altered genes and is widely regarded as a tumor suppressor in leukemogenesis [19]. Interestingly, RUNX1 exhibits context-dependent dual roles in solid tumors, functioning as either an oncogene or a tumor suppressor, with its pro-oncogenic activities attracting increasing attention in recent years [20–24]. Analyses of The Cancer Genome Atlas (TCGA) datasets further reveal that elevated RUNX1 expression is associated with poor clinical outcomes in CRC patients [25]. Despite these observations, the precise molecular functions and underlying mechanisms through which RUNX1 contributes to CRC initiation and progression remain incompletely understood.

The formation of the tumor microenvironment (TME) is regulated by tumor cells [26]. In this study, we investigated the role of RUNX1 in remodeling the TME of CRC. Elevated RUNX1 expression in CRC cells was found to correlate with reduced GITRL (GITR ligand) levels and an increased abundance of Treg cells within CRC tissues. Mechanistically, RUNX1 disrupts the GITRL-GITR signaling axis, which promotes Treg cell infiltration and suppresses CD8⁺ T cell activity. Consequently, RUNX1 expression sensitizes CRC cells to therapy with a GITR-activating antibody. Furthermore, we elucidated the underlying mechanism by which RUNX1 interacts with STAT1, inhibiting STAT1 dimerization and thereby suppressing STAT1-mediated transcription of GITRL. Collectively, our findings highlight the RUNX1/STAT1/GITRL axis as a promising therapeutic target for enhancing GITR antibody-based cancer immunotherapy.

## Results

### RUNX1 inhibits GITRL expression in CRC cells

**Fig. 1:**
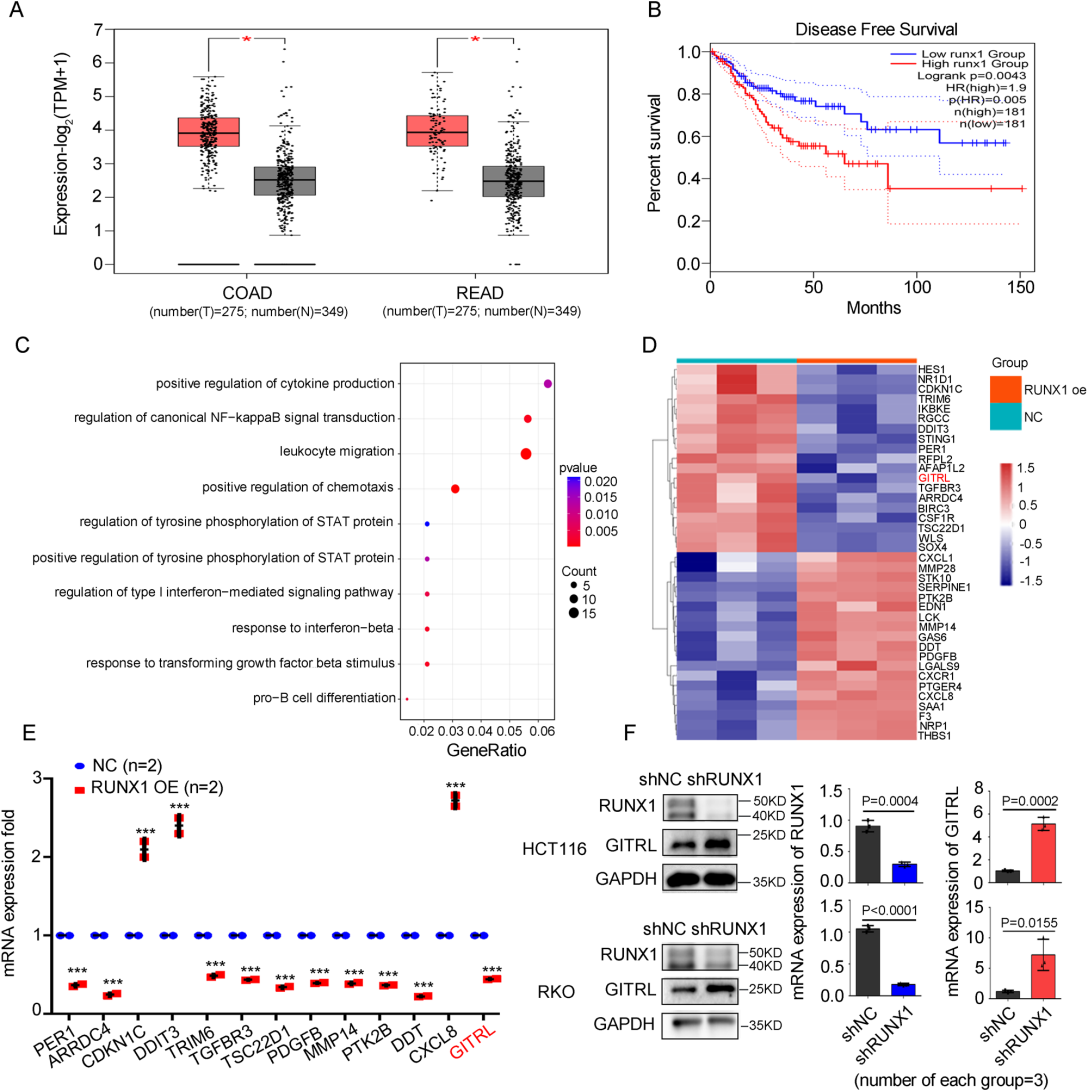
RUNX1 inhibits GITRL expression in CRC cells. **A** RUNX1 expression is significantly elevated in CRC tissues compared to normal tissues, as analyzed using the TCGA dataset via the GEPIA online platform. **B** Analysis of DFS data from GEPIA indicates that patients with low RUNX1 expression show a higher DFS rate compared to those with high RUNX1 expression. **C** GO-Biological Process enrichment analysis reveals that RUNX1 overexpression in HCT116 cells significantly enriches immune-related signaling pathways. **D** A heatmap illustrates distinct expression patterns of immune-related genes between RUNX1-overexpressing cells and wild-type control cells. **E** Quantitative RT-PCR confirms differential mRNA expression levels of selected immune-related genes in RUNX1-overexpressing cells vs control cells. **F** qRT-PCR and western blot analyses demonstrate efficient knockdown of RUNX1 and corresponding upregulation of GITRL at both mRNA and protein levels in HCT116 and RKO cells. Data are presented as mean ± SD from three independent experiments. ^*^P < 0.05; ^***^P < 0.001. Statistical analysis: Student’s *t* test (two-sided). COAD colorectal adenocarcinoma, READ rectum adenocarcinoma, T tumor, N normal, HR hazard ratio, NC negative control, sh shRNA, OE overexpression, n number, KD kilodalton.

Analysis of the TCGA database revealed that RUNX1 expression was significantly elevated in CRC tissues compared with adjacent non-tumorous tissues. Moreover, higher RUNX1 expression was associated with shorter disease-free survival (DFS) in CRC patients (*P* = 0.0043) (Fig. 1A, B), suggesting a potential role for RUNX1 in CRC progression. Consistent with this, prior studies have demonstrated that RUNX1 regulates migration in CRC cells [25]. Nevertheless, the function of RUNX1 within the immune microenvironment of CRC remains poorly characterized.

To investigate whether RUNX1 contributes to CRC development through modulation of the TME, we first performed RNA-seq and gene ontology (GO) analysis in HCT116 cells overexpressing RUNX1. This approach identified several immune-related pathways (Fig. 1C). Genes displaying significant expression differences within these pathways were selected and visualized in a heatmap (Fig. 1D). Subsequent qRT-PCR validation confirmed that the mRNA levels of 13 key genes were consistent with the RNA-seq data (Fig. 1E).

Among these 13 genes, TGFBR3, CXCL8, and GITRL are well-known immune-related factors. TGFBR3 is a cell-surface receptor that regulates diverse cellular processes, including proliferation, differentiation, migration, and apoptosis [27, 28]. CXCL8 is critically involved in neutrophil recruitment and activation [29, 30]. GITRL, by binding its receptor GITR, modulates the proliferation and activity of Treg cells and CD8⁺ T cells [31]. Given its immunoregulatory functions, we focused on GITRL and observed that RUNX1 knockdown in CRC cells led to increased GITRL expression at both mRNA and protein levels (Fig. 1F and Supplemental Fig. 1). Based on these findings, we sought to determine whether the RUNX1/GITRL axis influences the infiltration of Treg and CD8⁺ T cells in CRC.

### The GITRL-GITR signaling axis inhibits Tregs and activates CD8^+^ T cells

**Fig. 2:**
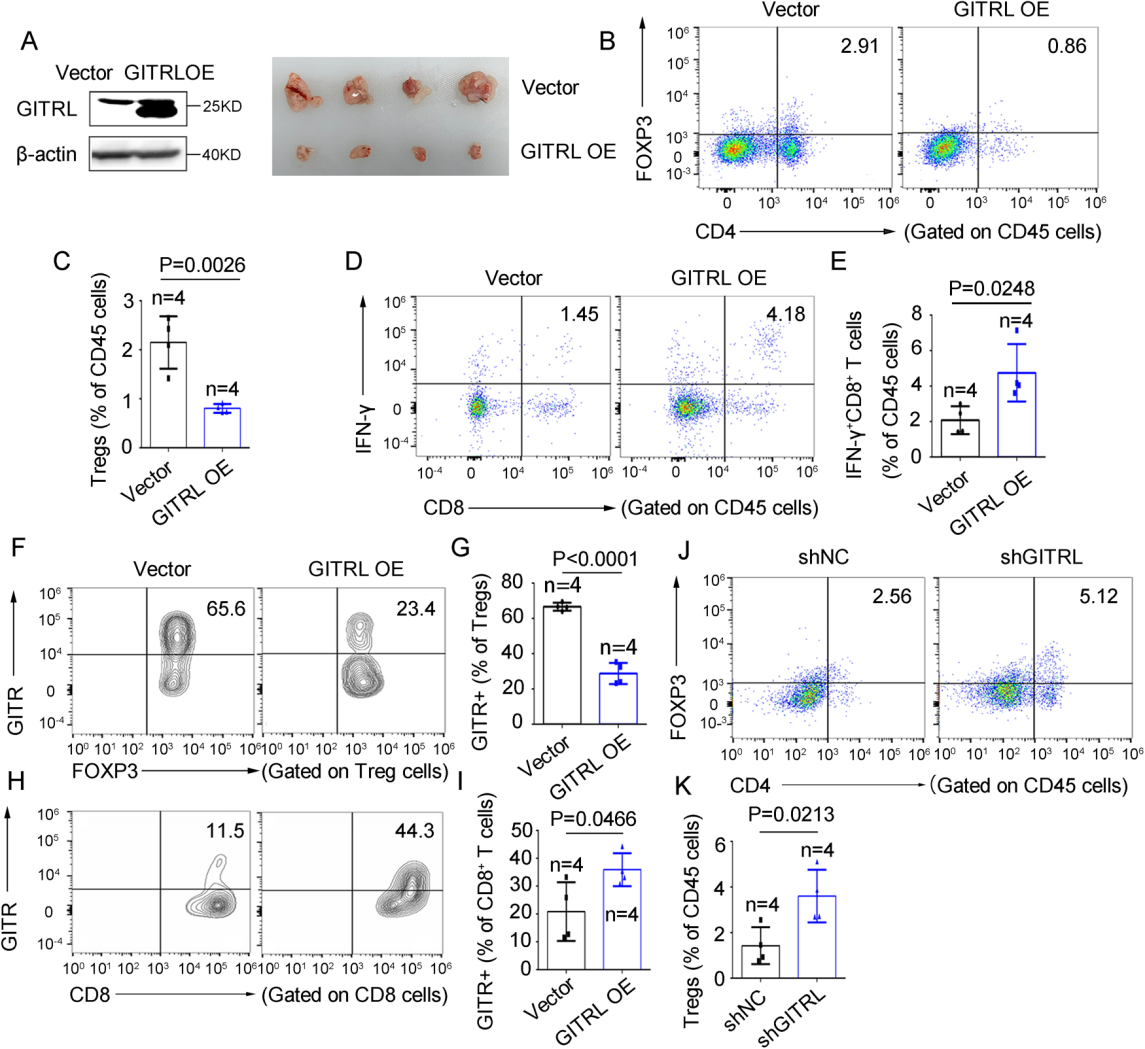
The GITRL-GITR signaling axis inhibits Tregs and activates CD8^+^ T cells. **A** Representative images of subcutaneous tumors derived from MC38 cells transduced with RUNX1-overexpressing or empty vector. **B**, **C** Proportions of Treg cells (FOXP3⁺CD4⁺) among CD45⁺ lymphocytes isolated from dissociated tumors, as determined by flow cytometry. **D**, **E** Proportions of IFN-γ⁺CD8⁺ T cells among CD45⁺ lymphocytes isolated from dissociated tumors, as determined by flow cytometry. **F**, **G** Expression of GITR on Treg cells analyzed by flow cytometry. **H**, **I** Expression of GITR on CD8⁺ T cells analyzed by flow cytometry. **J**, **K** Proportions of Treg cells (FOXP3⁺CD4⁺) among CD45⁺ lymphocytes isolated from dissociated tumors with GITRL silencing, as determined by flow cytometry. Animal experiments were performed twice with consistent results, and data are presented as mean ± SD from one representative experiment. Statistical analysis: Student’s *t* test (two-sided). NC negative control, sh shRNA, OE overexpression, n number, KD kilodalton. Vector: the empty vector as a negative control.

GITR is highly expressed on Treg cells and activated CD8⁺ T cells [31]. To investigate whether GITRL–GITR signaling modulates Treg and CD8⁺ T cell responses in CRC, MC38 cells with either GITRL overexpression or knockdown were subcutaneously engrafted into the flanks of C57BL/6J mice. Tumors were subsequently dissociated and analyzed for lymphocyte composition by flow cytometry. We observed that GITRL overexpression significantly suppressed tumor growth, accompanied by a reduced proportion of Treg cells (FOXP3⁺CD4⁺) and an increased proportion of activated CD8⁺ T cells (IFN-γ⁺CD8⁺) among CD45⁺ lymphocytes (Fig. 2A–E). Furthermore, GITR expression was decreased on Treg cells and increased on CD8⁺ T cells in these tumors (Fig. 2F–I). Conversely, knockdown of GITRL in MC38 cells resulted in enhanced infiltration of Treg cells (Fig. 2J, K). Collectively, these findings demonstrate that the GITRL–GITR axis plays a functional role in restraining Treg cell accumulation and promoting CD8⁺ T cell activation in CRC.

### RUNX1 promotes Tregs by inhibiting the GITRL‑GITR signaling axis

**Fig. 3:**
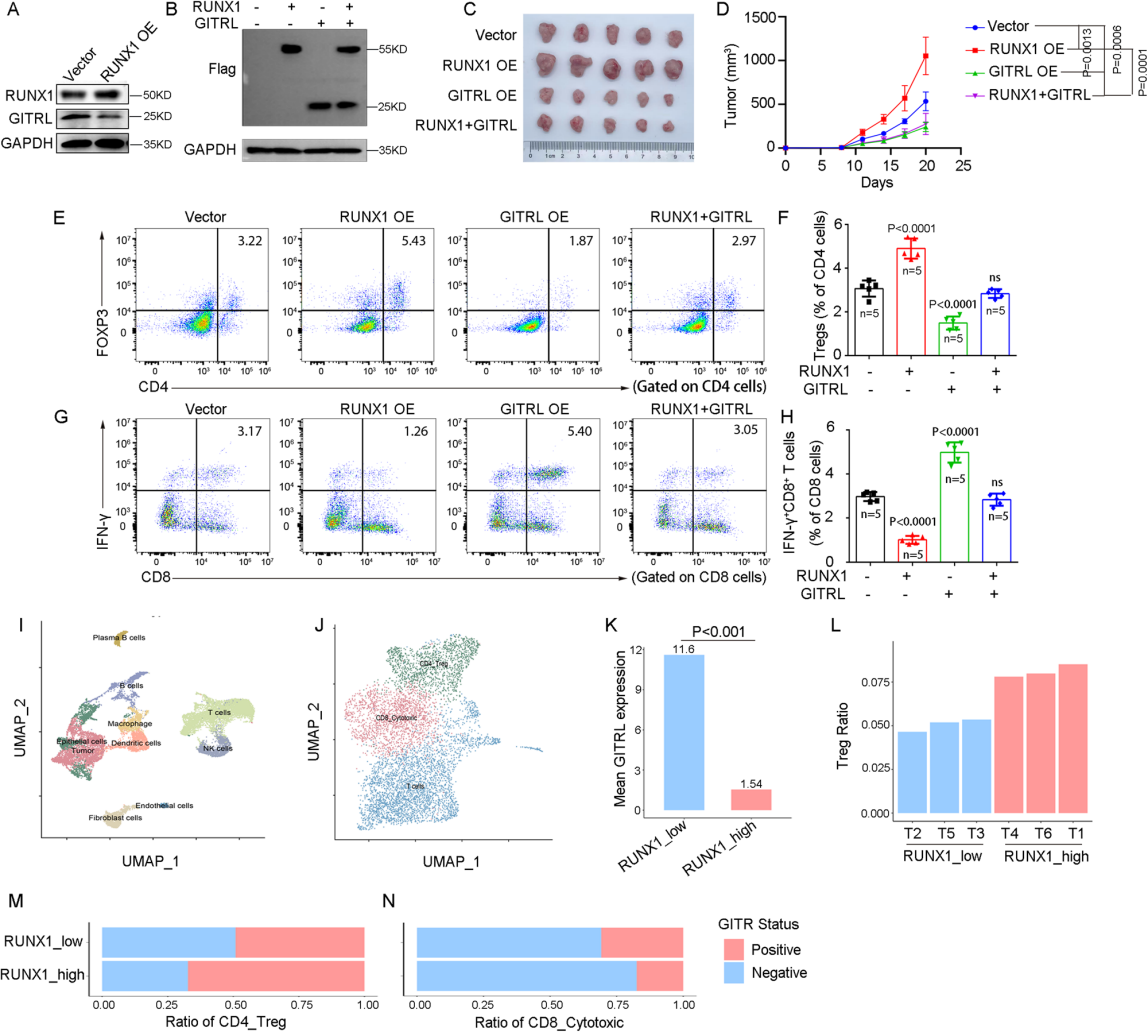
RUNX1 promotes Tregs by inhibiting the GITRL-GITR signaling axis. **A** Representative immunoblot analysis of GITRL expression in RUNX1-overexpressing MC38 cells. **B** Representative immunoblots of RUNX1-Flag or GITRL-Flag in MC38 cells. **C**, **D** Representative photograph (**C**) and growth curve (**D**) of subcutaneous tumors. **E**, **F** Flow-cytometric analysis of the proportion of Treg cells (FOXP3⁺CD4⁺) among CD4⁺ T cells within dissociated tumors. **G**, **H** Flow-cytometric analysis of the proportion of IFN-γ⁺CD8⁺ T cells among CD8⁺ T cells within dissociated tumors. **I**, **J** UMAP visualization of single-cell RNA-seq data from 6 CRC tissues, showing all cells (**I**) or T cells only (**J**). **K** Mean GITRL expression levels in RUNX1-high vs RUNX1-low groups. **L** Proportion of Treg cells among total tumor-infiltrating cells. **M** Percentage of GITR-expressing Treg cells among total Treg cells. **N** Percentage of GITR-expressing CD8⁺ T cells among total cytotoxic CD8⁺ T cells. Animal experiments were performed twice with consistent results, and data are presented as mean ± SD from one independent experiment. Statistical analysis: Student’s *t*-test (two-sided). OE overexpression. Vector: the empty vector as a negative control. T tissue, KD kilodalton.

Murine RUNX1 (mRUNX1) was overexpressed in MC38 cells, which resulted in significant downregulation of GITRL expression (Fig. 3A). Subcutaneous engraftment of these RUNX1-overexpressing MC38 cells into the flanks of C57BL/6J mice led to accelerated tumor growth. Importantly, this promotive effect was abrogated when GITRL was co‑overexpressed alongside RUNX1 (Fig. 3B–D). Flow cytometric analysis of dissociated tumor tissues revealed that RUNX1 overexpression increased the infiltration of Tregs (FOXP3⁺CD4⁺) and suppressed the frequency of activated CD8⁺ T cells (IFN‑γ⁺CD8⁺). These changes were reversed by concomitant GITRL overexpression (Fig. 3E–H).

Analysis of single‑cell RNA‑sequencing data from six human CRC specimens provided further clinical correlation (Fig. 3I, J): GITRL expression was significantly lower in RUNX1‑high compared to RUNX1‑low tumor cells (Fig. 3K). Additionally, RUNX1‑high tissues exhibited greater infiltration of Tregs (Fig. 3L) and an increased proportion of GITR⁺ Tregs, whereas the proportion of GITR⁺ CD8⁺ T cells was reduced (Fig. 3M, N). Collectively, these results demonstrate that RUNX1 promotes Treg accumulation and activity while impairing CD8⁺ T‑cell function, effects that are mediated through suppression of the GITRL–GITR signaling axis.

### RUNX1 expression correlates positively with Treg infiltration in CRC

**Fig. 4:**
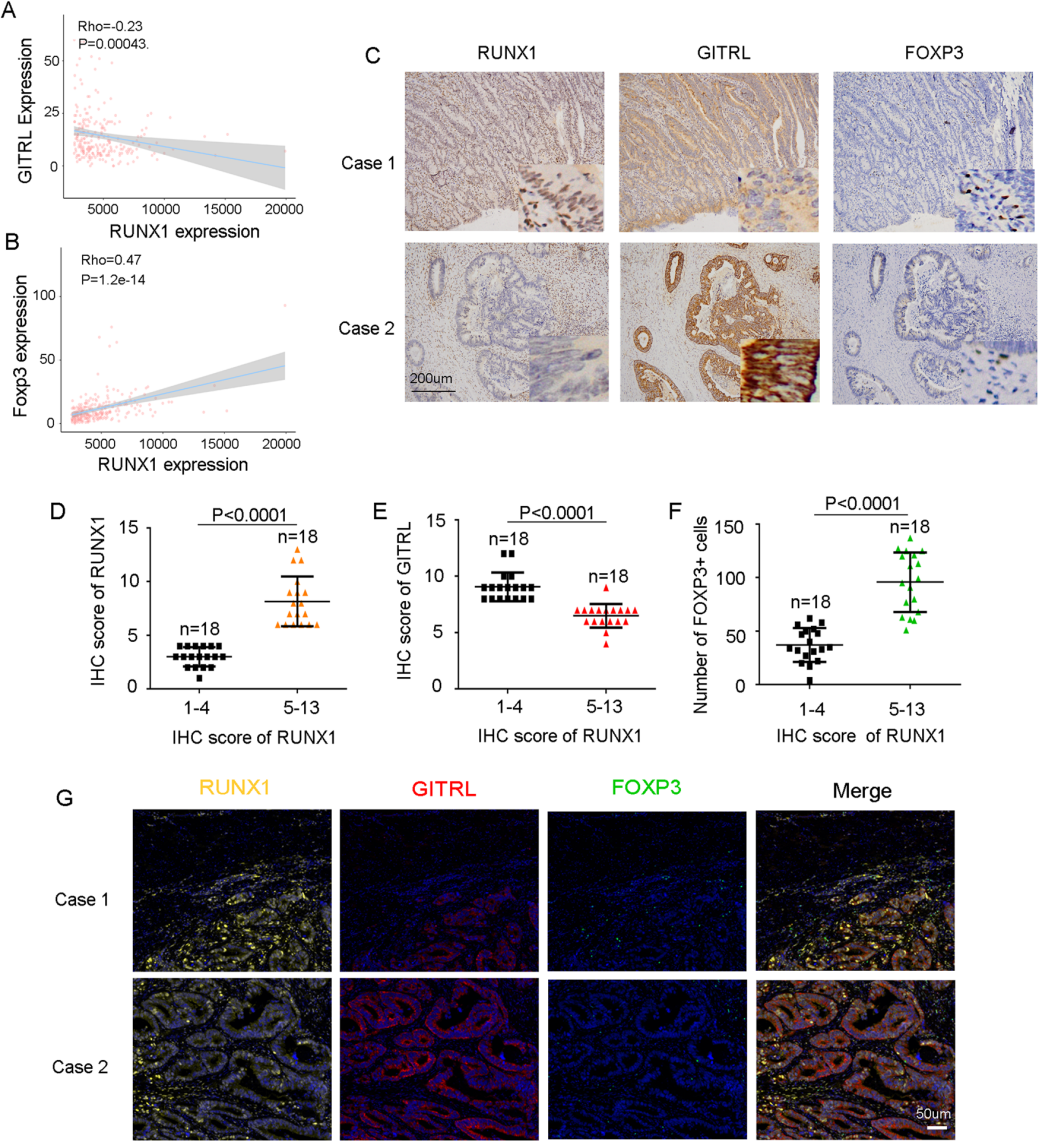
RUNX1 expression correlates positively with Treg infiltration in CRC. **A**, **B** Correlation analyses between RUNX1 and GITRL (**A**) or FOXP3 (**B**) expression based on the TCGA database. **C** Representative immunohistochemical staining of RUNX1, GITRL, and FOXP3 in CRC tissues. RUNX1 localizes to both the nucleus and cytoplasm of tumor cells, as well as of infiltrating lymphocytes. GITRL is primarily expressed in the cytoplasm of tumor cells, whereas FOXP3 is specifically expressed in regulatory T cells (Tregs). Scale bars: 200 μm. **D**–**F** Scatter plots showing the distribution of 36 CRC patients according to immunohistochemistry scores for RUNX1 or GITRL and the corresponding mean numbers of infiltrating FOXP3⁺ cells. FOXP3⁺ cells were counted in each 20× field of view, and the average number from five randomly selected fields was calculated for each sample. **G** Representative multiplex immunofluorescence images of three CRC tissue cases. Scale bars: 50 μm. Data presented are mean ± SEM. Statistical analysis: Student’s *t*-test (two-sided).

To further investigate the correlation between RUNX1 expression and GITRL in CRC cells, as well as the infiltration of Treg cells at the tissue level, we analyzed data from the TCGA database. This analysis revealed a significant negative correlation between RUNX1 and GITRL expression in CRC, whereas RUNX1 expression was positively correlated with Treg cell infiltration (Fig. 4A, B).

Subsequently, we collected 36 tumor tissue samples from CRC patients who had recently undergone surgery at our institution. Immunohistochemical (IHC) staining was performed on the 36 tissues to evaluate the expression of RUNX1, GITRL, and FOXP3. Based on IHC scores, samples were categorized into a RUNX1 high-expression group (score 5–13) and a RUNX1 low-expression group (score 1–4). In RUNX1-high tissues, GITRL expression was significantly lower than in RUNX1-low tissues (IHC score: 6.50 vs 9.06, *P* < 0.001). Conversely, the number of infiltrating Treg cells (FOXP3⁺) was markedly higher in RUNX1-high tissues compared with RUNX1-low tissues (95.78 vs 37.11, *P* < 0.001; Fig. 4C–F). Furthermore, multiplex immunofluorescence staining confirmed these expression patterns for RUNX1, GITRL, and FOXP3 in CRC tissues (Fig. 4G).

Collectively, these results demonstrate that RUNX1 expression is inversely correlated with GITRL in CRC cells and positively associated with Treg infiltration in CRC tissues. These findings suggest that RUNX1 and GITRL may serve as predictive biomarkers for the application of GITR agonistic antibodies in CRC treatment, potentially through modulating Treg cell infiltration.

### RUNX1 inhibits STAT1-mediated transcriptional activation of GITRL

**Fig. 5:**
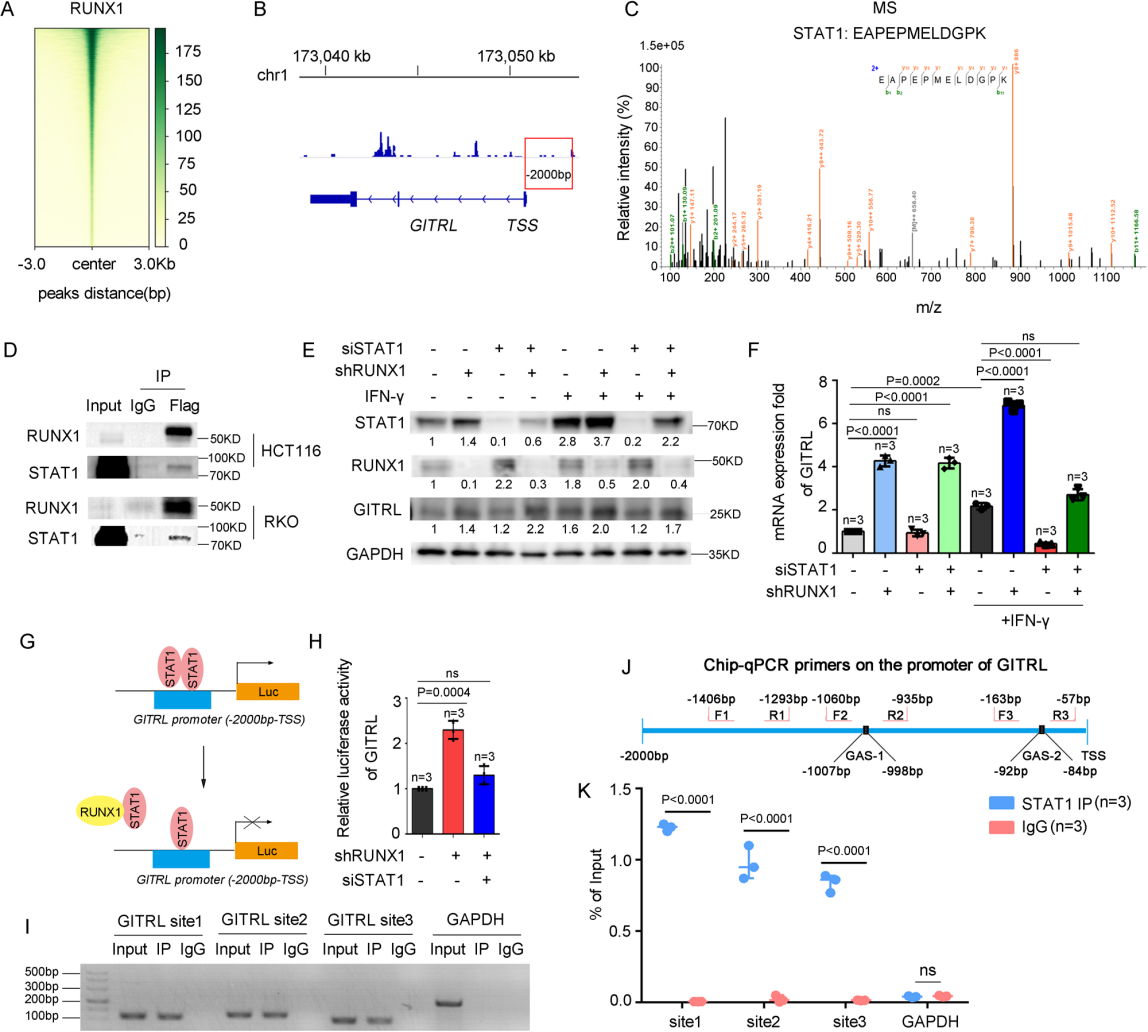
RUNX1 inhibits STAT1-mediated transcriptional activation of GITRL. **A** CUT&Tag density heat map of RUNX1 enrichment in HCT116 cells stably expressing RUNX1-flag, within 3 kb around TSS. Gene order is arranged from highest to lowest density. **B** CUT&Tag-seq data show that there are no RUNX1 peaks at the promoter region of GITRL. **C** Identification of STAT1 in the RUNX1-flag overexpressing HCT116 cells by MS. **D** Endogenous STAT1 was immunoprecipitated with exogenous RUNX1-flag in HCT116 and RKO cells. **E**, **F** Western blotting (**E**) and qPCR (**F**) analyses for the expression of STAT1, RUNX1, and GITRL in HCT116 cells with the silencing of RUNX1 or STAT1 in the presence or absence of 100 U mL^–1^ IFN-γ for 72 h. **G** A proposed model illustrating the regulation of GITRL’s luciferase activity by RUNX1 and STAT1. **H** HCT116 cells were cotransfected with the GITRL-Luc reporter and pRL-TK plasmid without IFN-γ stimulation, then subjected to a luciferase activity assay after 48 h. **I** ChIP-PCR of STAT1 abundance at the GITRL’s promoter in HCT116 cells after treatment with 100 U mL^–1^ IFN-γ for 24 h. **J** The amplicons of the three primers used for ChIP-qPCR at the GITRL promoter region. **K** ChIP-qPCR analysis of STAT1 abundance at the GITRL promoter in HCT116 cells after treatment with 100 U mL^–1^ IFN-γ for 24 h. HCT116 cells transduced with an empty vector are the negative control. Data presented are mean ± SD of three independent experiments. Statistical analysis: Student’s *t* test (two-sided). Kb kilobases, bp base pair, chr chromosome, TSS transcription start site, IP immunoprecipitation, ns not statistically, Luc luciferase, F forward primer, R reverse primer, sh shRNA, si siRNA, GAS gamma-activated sequence, n number, KD kilodalton.

To elucidate the mechanism by which RUNX1 downregulates GITRL expression, we first overexpressed RUNX1 in HCT116 cells and performed Cut&Tag sequencing to assess RUNX1 occupancy at the GITRL promoter (Fig. 5A). However, no direct binding of RUNX1 to the GITRL promoter region was detected, indicating that RUNX1 does not transcriptionally regulate GITRL directly (Fig. 5B and Supplemental Fig. 2 and Table 1). Given the observed reduction in GITRL mRNA levels upon RUNX1 expression, we hypothesized that RUNX1 might suppress GITRL transcription indirectly via other transcription factors. Proteomic analysis identified STAT1 as a candidate, as GITRL was enriched among STAT1‑regulated pathways in our earlier RNA‑seq data (Fig. 5C and Supplemental Fig. 3). Co‑immunoprecipitation (Co‑IP) confirmed a physical interaction between RUNX1 and STAT1 (Fig. 5D).

STAT1 is a key mediator of tumor‑immune signaling upon activation by interferon‑γ (IFN‑γ) [32]. Stimulation of CRC cells with IFN‑γ revealed that RUNX1 regulates GITRL mRNA and protein levels in a STAT1‑dependent manner (Fig. 5E, F and Supplemental Fig. 4). Notably, STAT1 knockdown alone had minimal effect on GITRL expression in the absence of IFN‑γ stimulation (Fig. 5E, F). Based on these findings, we propose the following model to explain the differential roles of STAT1 and RUNX1 under basal vs stimulated conditions:

In unstimulated CRC cells, GITRL is constitutively expressed at low levels. Under these basal conditions, STAT1 activity is limited, and potential compensatory heterodimerization among STAT family members may sustain basal GITRL expression, accounting for the little impact of STAT1 depletion. Following IFN‑γ stimulation, GITRL upregulation becomes strongly dependent on STAT1 homodimerization and transcriptional activity; thus, STAT1 knockdown markedly reduces GITRL expression. In contrast, loss of RUNX1 elevates GITRL even under weak stimulatory conditions, such as those mediated by low‑level IFN‑γ present in fetal bovine serum (FBS). We hypothesize that RUNX1 may normally function as a transcriptional repressor or a nuclear barrier that restricts STAT1 access to target promoters. In the absence of RUNX1, STAT1 nuclear translocation is facilitated, thereby enhancing GITRL expression even under suboptimal signaling. Thus, under weak or basal stimulation, RUNX1 acts as a gatekeeper that restrains STAT1‑mediated GITRL induction, whereas STAT1 itself becomes indispensable primarily under strong IFN‑γ signaling.

Further supporting this model, luciferase reporter assays (LRAs) demonstrated that RUNX1 knockdown significantly activated GITRL transcription in the absence of IFN‑γ stimulation, an effect that was abolished by concurrent STAT1 knockdown in CRC cells (Fig. 5G, H). Additionally, chromatin immunoprecipitation followed by quantitative PCR (ChIP‑qPCR) confirmed direct binding of STAT1 to the GITRL promoter upon IFN‑γ stimulation (Fig. 5I–K and Supplemental Fig. 5). Collectively, these results indicate that STAT1 directly activates GITRL transcription, and this activation is suppressed by RUNX1.

### RUNX1 inhibits IFN-γ-induced STAT1 dimerization

**Fig. 6:**
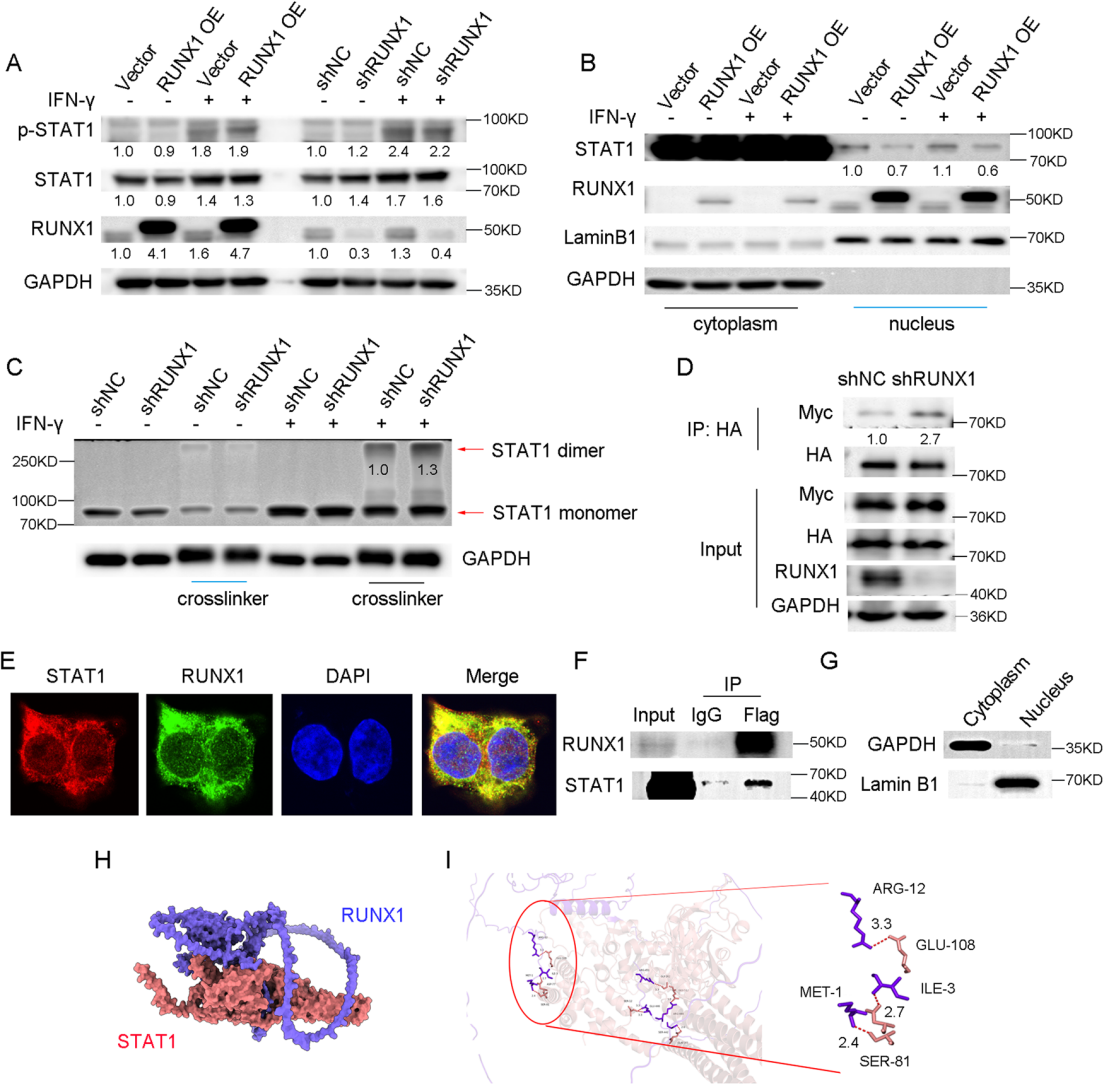
RUNX1 inhibits IFN-γ-induced STAT1 dimerization. **A** Western blot analysis of Tyr701-phosphorylated and total STAT1 protein levels in HCT116 cells following RUNX1 overexpression or knockdown, treated with or without 100 U·mL⁻¹ IFN-γ for 48 h. **B** Subcellular distribution of STAT1 protein in HCT116 cells overexpressing RUNX1, treated with or without 100 U·mL⁻¹ IFN-γ for 6 h, assessed by western blotting of nuclear and cytoplasmic fractions. **C** Detection of STAT1 dimer and monomer forms via western blotting in HCT116 cells with RUNX1 knockdown, treated with or without 100 U·mL⁻¹ IFN-γ for 6 h. **D** Co-immunoprecipitation (Co-IP) assay demonstrating that RUNX1 knockdown enhances STAT1-STAT1 interaction under stimulation with 100 U·mL⁻¹ IFN-γ for 48 h. HCT116 cells with RUNX1 knockdown were co-transfected with pcDNA3.1-HA-STAT1 and pcDNA3.1-Myc-STAT1, followed by immunoprecipitation with HA beads and immunoblotting with the indicated antibodies. **E** Confocal microscopy images showing cytoplasmic co-localization of STAT1 and RUNX1. Scale bars: 5 μm. **F**, **G** Co-IP assay indicating the interaction between RUNX1 and STAT1 in the cytoplasmic fraction. Lamin B1 and GAPDH served as nuclear and cytoplasmic reference proteins, respectively. **H**, **I** Predicted protein interaction sites between RUNX1 and STAT1 based on molecular docking analysis. HCT116 cells transduced with an empty vector were used as a negative control. IP immunoprecipitation, sh shRNA, NC negative control, OE overexpression, KD kilodalton.

Upon IFN-γ stimulation, STAT1 undergoes phosphorylation and dimerization, subsequently translocating into the nucleus to regulate the transcription of downstream target genes [32]. To determine whether RUNX1 affects STAT1 activation, we first examined its potential role in STAT1 phosphorylation; however, western blot analysis showed no detectable alteration in phosphorylated STAT1 levels upon RUNX1 modulation (Fig. 6A). We then overexpressed RUNX1 in CRC cells and performed cytoplasmic and nuclear fractionation experiments, which revealed a reduction of STAT1 protein within the nuclear compartment, suggesting that RUNX1 impairs STAT1 nuclear entry (Fig. 6B). Further experiments demonstrated that RUNX1 also suppresses IFN-γ-induced STAT1 dimerization (Fig. 6C, D and Supplemental Fig. 6–8). Moreover, fluorescence colocalization and co-immunoprecipitation assays confirmed an interaction between RUNX1 and STAT1 in the cytoplasm (Fig. 6E–G). Protein-protein docking analysis provided additional support for a direct interaction between these two proteins (Fig. 6H, I). Collectively, these findings indicate that RUNX1 interacts with STAT1 in the cytoplasm and thereby inhibits IFN-γ-mediated STAT1 dimerization.

### RUNX1 sensitizes CRC cells to GITR agonistic antibody

**Fig. 7:**
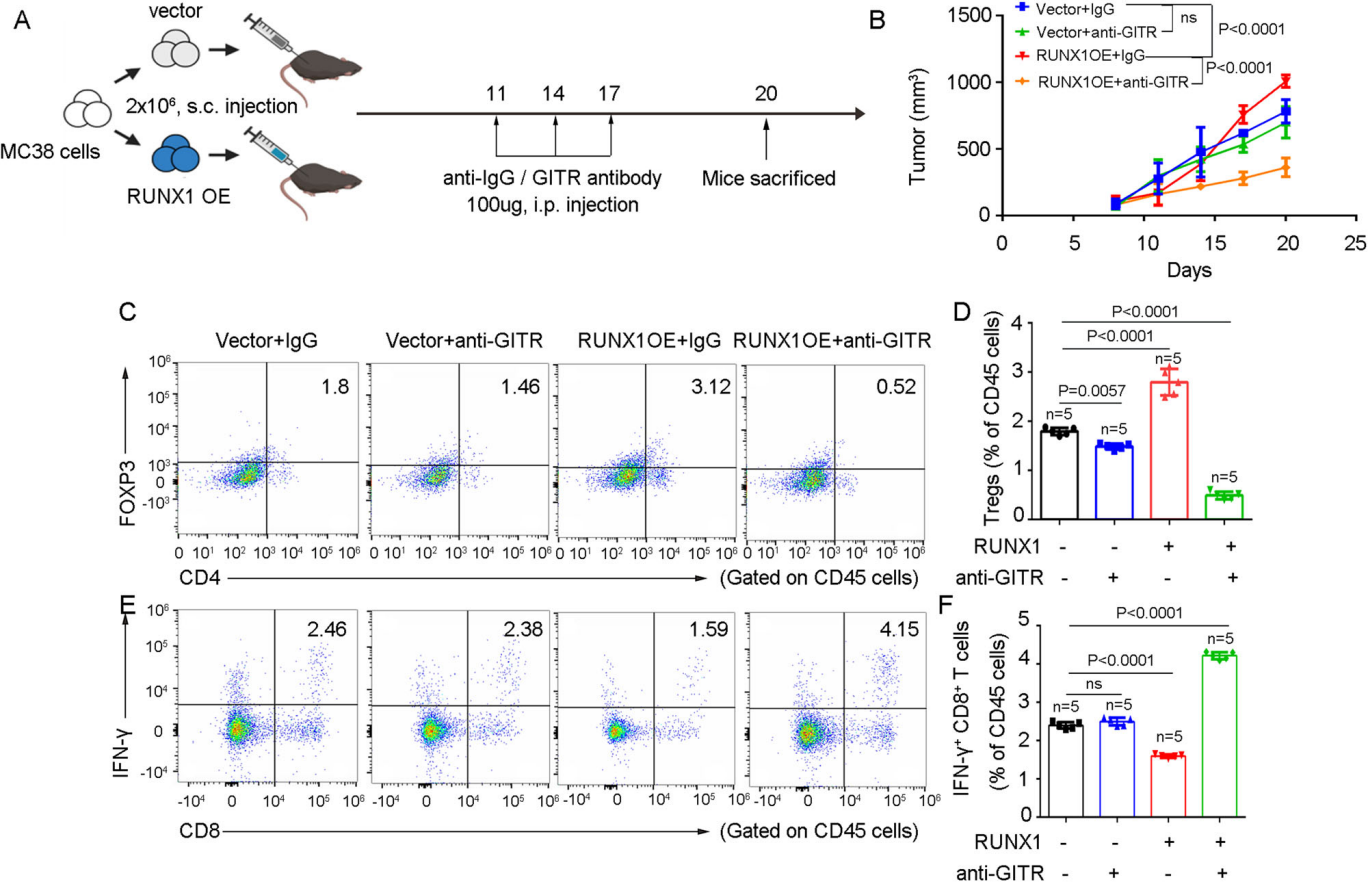
RUNX1 sensitizes CRC cells to GITR agonistic antibody. **A** Schematic representation of the ICB therapeutic regimen. MC38 cells (vector- or RUNX1-overexpressing) were subcutaneously inoculated into mice. When tumors became established, mice received intraperitoneal injections of anti-mouse GITR antibody or were left untreated on days 11, 14, and 17. Tumor growth was monitored throughout the study. **B** Growth curves of subcutaneous tumors over time. **C**, **D** Proportions of Tregs (FOXP3⁺CD4⁺) among CD45⁺ lymphocytes isolated from dissociated tumors. **E**, **F** Proportions of IFN-γ⁺CD8⁺ T cells among CD45⁺ lymphocytes isolated from dissociated tumors. Animal experiments were repeated twice with consistent trends; data shown are the mean ± SD from one independent experiment. Statistical analysis: Student’s *t* test (two-sided). OE overexpression. Vector: negative control. s.c subcutaneously, i.p intraperitoneally, anti-GITR GITR agonist, n number.

Previous studies have reported that GITR agonistic antibodies can promote the expansion of CD8⁺ T cells and suppress Treg cell infiltration, thereby providing a rationale for GITR-targeted immunotherapy in CRC patients [33]. Given our finding that RUNX1 overexpression in CRC cells leads to an increase in GITR⁺ Tregs, we sought to investigate whether RUNX1-overexpressing CRC cells exhibit enhanced sensitivity to GITR agonistic antibody treatment. To test this, MC38 cells were subcutaneously engrafted into the flanks of C57BL/6J mice, followed by intraperitoneal administration of GITR agonistic antibody (100 µg/mouse) according to the schedule illustrated in Fig. 7A. We observed that RUNX1 overexpression in MC38 cells accelerated tumor growth; however, this effect was significantly inhibited upon concomitant treatment with the GITR antibody (Fig. 7B). Of note, GITR antibody treatment alone did not suppress tumor growth. Flow cytometric analysis of TILs revealed that RUNX1 overexpression increased the proportion of Treg cells (FOXP3⁺CD4⁺) and reduced the frequency of activated CD8⁺ T cells (IFN-γ⁺CD8⁺) within tumors—effects that were reversed by GITR agonist administration (Fig. 7C–F). Collectively, these results indicate that RUNX1 overexpression renders tumor cells more sensitive to GITR agonistic antibody therapy.

## Discussion

Our study reveals that elevated RUNX1 expression in CRC cells is associated with decreased GITRL expression and an increased abundance of Treg cells within CRC patient tumors. Functional experiments demonstrate that RUNX1 overexpression attenuates the GITRL–GITR signaling axis, thereby promoting Treg cell infiltration and suppressing CD8⁺ T cell activity. Consequently, RUNX1-high CRC tumors exhibit enhanced sensitivity to treatment with a GITR agonistic antibody. Mechanistically, RUNX1 interacts with STAT1 and inhibits its dimerization and transcriptional activation of GITRL. These findings highlight the RUNX1/GITRL pathway as a promising therapeutic target for potentiating GITR agonist-based immunotherapy in CRC.

Our understanding of the precise role and underlying mechanisms of RUNX1 in CRC initiation and progression remains incomplete. Recent studies indicate that RUNX1 enhances the migratory and invasive capacities of CRC cells by activating the Wnt/β-catenin signaling pathway. Moreover, RUNX1 can directly interact with β-catenin and subsequently bind to promoter and enhancer regions of the *KIT* gene to promote its transcription [25]. Nevertheless, whether RUNX1 influences the TME in CRC is still unclear. In this study, we identified downstream targets of RUNX1 via RNA-seq and demonstrated that RUNX1 suppresses the expression of *GITRL*. The receptor GITR is primarily expressed on Treg cells and activated CD8⁺ T cells [31]. Thus, it is of considerable interest to determine whether RUNX1 modulates Treg cell infiltration and CD8⁺ T-cell activity in CRC through the GITRL–GITR signaling axis.

Initial studies indicated that GITRL was predominantly localized in non-lymphoid tissues. However, subsequent research has demonstrated that GITRL is constitutively expressed by antigen-presenting cells (APCs) in secondary lymphoid organs, distinguishing it from most other ligands of the TNFR family [31]. More recently, it was shown that GITRL is highly expressed in hepatic progenitor cells, where it promotes proliferation by activating the epithelial-mesenchymal transition (EMT) pathway and enhancing phosphorylation of ERK1/2 and Akt via interaction with ANXA2 [34]. Nevertheless, whether GITRL expressed by CRC cells influences the recruitment or retention of GITR-positive immune cells in the TME remains unclear.

As reported in prior research, the analysis of lymphocytes from resected tumors, adjacent tissues, and peripheral blood mononuclear cells (PBMCs) of 132 CRC patients indicated that GITR agonistic antibody can promote CD8^+^ T cell expansion and inhibit Treg cell infiltration, thereby supporting the therapeutic targeting of GITR in CRC [33]. Moreover, studies have shown that GITR activity on Tregs involves Foxp3 destabilization and Treg depletion, while GITR activity on CD8^+^ T cells enhances cytotoxic T lymphocyte responses via NF-κB induction [35].

Given that the active structure of the GITR agonist resembles the endogenous GITR ligand (GITRL), we sought to determine whether tumor cell-expressed GITRL could mimic the effects of the GITR agonist. To this end, we overexpressed GITRL in mouse CRC cells (MC38) and established subcutaneous tumors in C57BL/6 mice. Consistent with our hypothesis, flow cytometry analysis demonstrated that GITRL overexpression reduced the proportion of Treg cells and increased the frequency of activated CD8^+^ T cells among TILs (Fig. 2B–E). Additionally, GITRL expression decreased the proportion of GITR-positive Tregs while increasing GITR-positive CD8^+^ T cells, suggesting that tumor-derived GITRL can suppress GITR^+^ Tregs and promote GITR^+^ CD8^+^ T cells (Fig. 2F–I).

It is well-established that Treg cells constitutively express high levels of GITR, whereas CD8^+^ T cells express low basal levels that are upregulated upon activation [31]. Our data align with this pattern: in mouse models, GITR expression was markedly higher on Tregs than on CD8^+^ T cells (65.6% vs 11.5%; Fig. 2F–I), a finding corroborated in samples from six CRC patients (Fig. 3M, N). This supports the notion that Tregs are key cellular targets in the modulation of GITR agonist.

Therefore, when RUNX1 suppresses GITRL expression in CRC cells, the ligand-mediated modulation of Tregs and CD8^+^ T cells is diminished, resulting in increased GITR^+^ Tregs and decreased GITR^+^ CD8^+^ T cells. Under these conditions, the GITR agonistic antibody more effectively depletes GITR^+^ Tregs, alleviates their suppressive effect on CD8^+^ T cells, and consequently enhances the sensitivity of RUNX1-overexpressing CRC cells to GITR-targeted immunotherapy.

The STAT1 signaling pathway plays a critical role in regulating cell growth, differentiation, homeostasis, and immune responses [36]. Substantial evidence indicates that STAT1 exhibits tumor-suppressive properties in various cancers, with reduced STAT1 expression and activation often correlating with tumor progression [37]. For example, STAT1 has been shown to inhibit ErbB2/Neu-mediated tumorigenesis in transgenic mouse models [38]. However, despite the established tumor-suppressive functions of STAT1, several studies have associated elevated STAT1 activity with poor clinical outcomes in certain malignancies [39]. In breast cancer, STAT1 activation by CD95 (Fas)—a receptor that induces apoptosis via caspase cascade initiation—promotes STAT1-dependent mammosphere formation [40]. Moreover, epigallocatechin gallate (EGCG), a major bioactive component of green tea and a known STAT1 inhibitor, has been demonstrated to suppress proliferation and induce apoptosis in luminal A breast cancer cells [41, 42]. Therefore, whether STAT1 acts as a promoter or an inhibitor of tumor growth may depend on contextual factors within the TME, warranting further investigation.

In this study, we demonstrate that STAT1 exerts an anti-tumor effect in CRC cells by transcriptionally activating GITRL expression, thereby promoting antitumor immunity. This STAT1-mediated activation of GITRL is inhibited by RUNX1, which interacts with STAT1 in the cytoplasm to impair its dimerization and subsequent activation. In summary, our study reveals the new function and mechanism of the RUNX1/STAT1/GITRL axis in regulating CRC immunity, and provides the new biomarkers of the GITR agonistic antibody for the treatment of CRC.

## Materials and methods

### Clinical specimens and experimental animals

All patients enrolled in this study were pathologically diagnosed with CRC. Tumor specimens were obtained from Guangdong Provincial People’s Hospital. The study protocol was approved by the Institutional Review Board of Guangdong Provincial People’s Hospital (approval no. KY2024-849-01), and written informed consent was obtained from all participants for the use of their clinical data.

Five- to six-week-old male C57BL/6J mice were purchased from the Guangdong Medical Laboratory Animal Center (Guangzhou, China). Mice were housed under specific pathogen-free (SPF) conditions at Daoke Medical & Pharmaceutical Company (Guangzhou, China). All animal procedures were performed in a randomized design and were approved by the Institutional Review Board of Guangdong Provincial People’s Hospital (Approval No. KY2024-849-01) in compliance with relevant ethical guidelines.

### Cell lines and reagents

The human CRC cell lines HCT116 and RKO were obtained from the American Type Culture Collection (ATCC). The murine MC38 cell line was acquired from the Shanghai Cell Bank of the Chinese Academy of Sciences. All cells were maintained in Dulbecco’s Modified Eagle Medium (DMEM; PM150210, Procell) or RPMI 1640 medium (PM150110, Procell), supplemented with 10% FBS (F0193, Sigma) and 1% penicillin-streptomycin. Cultures were incubated at 37 °C in a humidified atmosphere containing 5% CO₂. Recombinant human IFN-γ (300-02, Peprotech) was reconstituted and stored according to the manufacturer’s instructions.

### Plasmid construction and virus infection

To generate the constructs for this study, the full-length human or mouse RUNX1 cDNA (reference sequences NM_001001890.3 or NM_001111022.2) was cloned into multiple expression backbones, including pLVX-hRunx1(V2)-3×FLAG-Puro, pcDNA3.1(+)-hRUNX1(V2)-3×FLAG-linker-mCherry, or pLVX-mRunx1(V2)-3×FLAG-Puro. Similarly, the full-length mouse GITRL cDNA (reference sequence NM_183391.4) was inserted into pCDH-CMV-mGITRL-EF1-Puro-3×Flag. All plasmid constructs were generated by HanYi Biosciences (Guangzhou, China). For gene knockdown, shRNA sequences targeting human RUNX1, human STAT1, and mouse GITRL were designed and cloned into the pLKO.1-shRNA-puro vector (also provided by HanYi Biosciences). All shRNA sequences are listed in Supplementary Table 2.

For lentivirus production, the recombinant lentiviral vectors were transfected into 293 T packaging cells using EZ Trans transfection reagent (AC04L099, Life iLab). At 48 h post-transfection, the viral supernatant was collected, filtered through a 0.45 μm nitrocellulose membrane, and used to infect target cells. Stable cell lines were selected with puromycin at 1.0 or 8.0 μg/mL for 3 days beginning 48 h after infection.

### RNA interference

Cells were seeded in culture plates and transfected with the indicated siRNAs using Lipofectamine 6000 (C0526, Beyotime) according to the manufacturer’s instructions. All siRNAs were obtained from HanYi Biosciences, and their sequences are provided in Supplementary Table 2.

### Quantitative real-time PCR (qRT-PCR) and RNA-Seq

Total RNA was isolated from cells with Trizol reagent (AG21102, AG). For qRT-PCR analysis, RNA was reverse-transcribed into cDNA using a reverse transcription reagent (AG11706, AG). cDNA was subsequently amplified and quantified with a quantitative PCR kit (AG11701, AG) on a LightCycler 480 Ⅱ Real-Time PCR system (Roche, 1610). For RNA-Seq, libraries were prepared according to the manufacturer’s instructions using the NEBNext Ultra™ RNA Library Prep Kit (E7490, NEB). Sequencing was performed on the Illumina NovaSeq 6000 platform by Novogene. Each sample yielded approximately 6 GB of clean data. Clean reads were aligned to the human reference genome GRCh38 (hg38) using the STAR aligner. All primer sequences used for qRT-PCR are listed in Supplementary Table 3.

### Western blotting and co-immunoprecipitation (Co-IP)

Western blot analysis was performed according to standard protocols. The following antibodies were used for immunoblotting: anti-GITRL (NBP1-77240, Novus; 1:1000), anti-RUNX1 (25315-1-AP, Proteintech; 1:1000), anti-STAT1 (10144-2-AP, Proteintech; 1:1000), anti-phospho-STAT1 (Tyr701) (28979-1-AP, Proteintech; 1:1000), anti-HA tag (51064-2-AP, Proteintech; 1:1000), anti-Myc tag (60003-2-Ig, Proteintech; 1:1000), anti-Lamin B1 (12987-1-AP, Proteintech; 1:1000), and anti-GAPDH (60004-1-Ig, Proteintech; 1:1000). Protein bands were visualized with Super ECL Detection Reagent (36208ES76, YEASEN).

For co-immunoprecipitation (Co-IP), cell lysates were incubated overnight at 4 °C with either Flag-tagged magnetic beads (P2181S, Beyotime) or HA-tagged magnetic beads (P2185S, Beyotime). The immunoprecipitated complexes were washed three times with mild RIPA lysis buffer and subsequently analyzed by Western blotting.

Band intensities were quantified using ImageJ software, and the gray values of each target protein band were normalized to those of GAPDH.

### In vivo studies

Five- to six-week-old male C57BL/6J mice were obtained from the Guangdong Medical Laboratory Animal Center (Guangzhou, China). All mice were housed under SPF conditions at Daoke Medical & Pharmaceutical Company in Guangzhou. Animals were randomly allocated to experimental groups using a computer-generated randomization sequence. MC38 cells (2 × 10⁶) were subcutaneously inoculated into the left flank of each mouse. Tumor growth was monitored every four days post-inoculation until the tumor volume reached approximately 1000 mm³. Tumor volume measurements were performed by investigators blinded to group allocation. To evaluate the effect of the GITR agonistic antibody (BE0063, BioXcell), tumor-bearing mice were administered intraperitoneal injections of either control IgG or the anti-GITR agonistic antibody (100 µg per mouse) at designated time points. Tumors were subsequently excised for further analysis.

For analysis of TILs, excised tumors were dissociated and passed through a cell strainer to generate single-cell suspensions. Cells were stained with the following fluorescently conjugated antibodies for flow cytometry: anti-CD45 (1:200 dilution, eBioscience, cat. 56-0451-80), anti-CD4 (1:200 dilution, eBioscience, cat. 48-0041-80), anti-CD8 (1:200 dilution, eBioscience, cat. 17-0083-81), anti-GITR (1:200 dilution, eBioscience, cat. 12-5874-82), anti-IFN-γ (1:100 dilution, eBioscience, cat. 12-7311-82), and anti-FOXP3 (1:100 dilution, eBioscience, cat. 45-5773-80).

### Flow cytometry

Single-cell suspensions were prepared from mouse spleens for use as control samples. These were divided into a negative control tube and individual single-color fluorescence staining tubes to facilitate voltage adjustment and gating. The gating strategy is illustrated in Supplemental Figure 9. Each flow cytometry experiment was performed with four to five replicates.

For surface antigen staining, the following fluorophore-conjugated antibodies were used: anti-CD45, anti-CD4, anti-CD8b, and anti-GITR. Prior to intracellular cytokine staining, cells were stimulated for 4–6 h at 37 °C with PMA (1 µM, HY-18739, MCE), ionomycin (1 µM, HY-13434A, MCE), and brefeldin A (BFA, 1 µM, HY-16592, MCE). Intracellular staining was performed using the Transcription Factor Staining Buffer Kit (IC001-100, BD) together with antibodies against Foxp3 and IFN-γ.

Stained samples were analyzed on a CytoFLEX S flow cytometer, and data were processed with FlowJo software (version 10.3).

### Immunohistochemistry and evaluation

Tissue sections were deparaffinized in dimethylbenzene and rehydrated through a graded alcohol series. Endogenous peroxidase activity was quenched by treating the sections with 0.3% hydrogen peroxide for 15 min. Antigen retrieval was performed by boiling the slides in EDTA buffer (pH 8.0) using a pressure cooker (1400 W) for 8 min. Nonspecific binding was blocked by incubating the sections with 10% normal goat serum for 1 h at room temperature. Subsequently, the slides were incubated overnight at 4 °C in a humidified chamber with primary antibodies against RUNX1 (1:400, 25315-1-AP, Proteintech), GITRL (1:1500, 23899-1-AP, Proteintech), or FOXP3 (1:1500, UM870140, Origene). Following primary antibody incubation, the sections were treated with a horseradish peroxidase (HRP)-conjugated goat anti-rabbit/mouse IgG H&L secondary antibody (bs-0295G-HRP, Bioss) for 1 h at room temperature. Immunoreactivity was visualized using a 3,3′-diaminobenzidine (DAB) substrate.

Immunoreactivity scores (IRS) for RUNX1, GITRL, and FOXP3 were assessed independently by two pathologists who were blinded to the sample information. Staining intensity was graded as follows: 0 (negative), 1 (weak), 2 (moderate), or 3 (strong). The percentage of positive cells was scored on a scale of 0 (<10%), 1 (11–25%), 2 (26–50%), 3 (51–75%), or 4 (76–100%). The total IRS, ranging from 0 to 12, was calculated as the product of the intensity score and the percentage score. For RUNX1, immunohistochemistry (IHC) scores were derived from the sum of nuclear and cytoplasmic staining, yielding a range of 0 to 24. The final IRS for each sample represented the mean value from both pathologists. In cases where the discrepancy between the two scores exceeded 3, the specimen was re-evaluated jointly to reach a consensus score.

### Multiplex immunofluorescence staining

Multiplex immunofluorescence staining was performed to evaluate the expression of RUNX1 and GITRL in tumor cells, along with the presence of FOXP3-expressing Tregs in human CRC tissues. A five-color multiplex immunohistochemistry kit (KR Pharmtech, Shanghai, China) was employed according to the manufacturer’s instructions. The primary antibodies used were as follows: anti-FOXP3 (1:100 dilution, Ab191416, Abcam), anti-GITRL (1:1500 dilution, 23899-1-AP, Proteintech), and anti-RUNX1 (1:100 dilution, 25315-1-AP, Proteintech). Whole-slide scanning was performed at 20× magnification using the KR-HT5 system. Subsequent image analysis was carried out with inForm 2.4.0 software (KR Pharmtech, Shanghai, China).

### High-throughput cleavage under targets and tagmentation (CUT&Tag)

CUT&Tag analysis was performed as previously described [43]. An anti-Flag primary antibody (F1804, Sigma) was used at a dilution of 1:50. Raw sequencing data have been deposited in the Supplemental Material.

### Mass spectrometry (MS) analysis

Briefly, HCT116 cells were transfected with plasmids encoding Flag-tagged RUNX1. After transfection, cells were harvested and lysed in RIPA lysis buffer (KGP704-100, KEYGEN BIOTECH) supplemented with protease inhibitor cocktail (P001, NCM) and phosphatase inhibitor cocktail (P003, NCM) to prevent enzymatic degradation. Lysates were centrifuged at 13,500×*g* to remove cellular debris, and the resulting supernatants were incubated with Flag-conjugated beads (P2181S, Beyotime) for 4 h at 4 °C to immunoprecipitate Flag-tagged RUNX1 and its interacting proteins. The beads were then washed three times with a mild RIPA lysis buffer to eliminate nonspecifically bound proteins. Proteins bound to the beads were separated by sodium dodecyl sulfate–polyacrylamide gel electrophoresis (SDS-PAGE) and subsequently analyzed by MS. MS analysis was performed by Shanghai Applied Protein Technology Co., Ltd. (Shanghai, China).

### The LRA

Briefly, cells were seeded in 12-well plates at a density of 5 × 10⁵ cells per well. Subsequently, the cells were co-transfected with a promoter-luciferase reporter plasmid (0.75 μg) and a pRL-TK plasmid (0.75 μg) expressing *Renilla* luciferase as an internal control. Luciferase activity was measured 48 h post-transfection using a dual-luciferase assay kit (FR201-01-v2, TransGen). In this experiment, no IFN-γ stimulation was applied. The promoter fragment of GITRL spanned from –2000 bp to the transcription start site (TSS), and no motif mutagenesis was performed.

### ChIP-qPCR

For chromatin immunoprecipitation (ChIP) analysis, chromatin was isolated from HCT116 cells treated with 100 U mL^−1^ IFN-γ for 24 h. Chromatin was fragmented using a SCIENTZ-IID sonicator. Immunoprecipitation was performed with an anti-STAT1 antibody (1:50 dilution, #9172, Cell Signaling Technology) according to the manufacturer’s instructions for the ChIP detection kit (Bes5001, BersinBio). Immunoprecipitated DNA fragments were then used as templates for qPCR with ChamQTM Universal SYBR qPCR Master Mix (Q711-02, Vazyme) on a CFX ConnectTM Real-Time System (Bio-Rad). Primer sequences used in this assay are provided in Supplementary Table 4.

### Cytoplasmic and nuclear fractionation

Cells were seeded in 6-cm culture dishes and transfected with plasmids encoding 3×Flag-tagged RUNX1. Following transfection, IFN-γ was administered at a concentration of 100 U·mL⁻¹. Forty-eight hours post-transfection, cytoplasmic and nuclear fractions were isolated using a separation kit (SC-003, Invent). Protein lysates from each fraction were subsequently analyzed by western blotting.

### Chemical cross-linking

Briefly, cells were seeded in 6-well plates at a density of 1 × 10⁶ cells per well. After attachment, cells were treated with ice-cold phosphate-buffered saline (PBS, pH 7.0) containing 0.1% Triton X-100 and protease inhibitor cocktails (P001, NCM) for 15 min on ice to lyse the cells. The resulting protein lysates were then incubated with the cross-linker BS3 (ab145612, Abcam) at a final concentration of 2.5 mM for 1 h at room temperature. To terminate the cross-linking reaction, Tris-HCl (1 M, pH 7.5) was added, followed by incubation for an additional 15 min at room temperature. Finally, the cross-linked products were analyzed by Western blotting.

### Immunofluorescence confocal assay

Cells were seeded onto confocal dishes and fixed with 4% paraformaldehyde, followed by permeabilization with 0.2% Triton X-100. After three washes to remove residual reagents, nonspecific binding sites were blocked by incubation with goat serum for 1 h at room temperature. Subsequently, the cells were incubated overnight at 4 °C with the following primary antibodies: anti-RUNX1 (1:100 dilution, 25315-1-AP, Proteintech) and anti-STAT1 (1:100 dilution, M1407-1, HUABIO). Following three additional washes, the cells were incubated for 1 h at room temperature with fluorescent secondary antibodies in the dark, using Goat Anti-Rabbit IgG H&L (Alexa Fluor® 488) and Goat Anti-Mouse IgG H&L (Alexa Fluor® 594). After another three washes, nuclei were stained with DAPI for 15 min. Upon completion of the staining procedure, immunofluorescence images were acquired using a Zeiss LSM900 confocal microscope.

### Protein-protein docking analysis

The amino acid sequences of RUNX1 (accession number NP_001001890.1) and STAT1 (accession number NP_001371820.1) were retrieved from the NCBI database. The three-dimensional structures of these proteins and their potential interaction interface were subsequently predicted using the AlphaFold3 web server. The resulting structural model was visualized with PyMOL software to identify and depict key hydrogen-bonding residues at the protein-protein interface.

### Single-cell RNA sequencing data analysis

Single-cell RNA sequencing (scRNA-seq) data of CRC were retrieved from the GEO database (GSE231559; PMID: 37768068), comprising six tumor samples and three adjacent normal tissues. Data preprocessing and downstream analyses were performed using the “Seurat” package (version 4.2.0) (PMID: 37231261). Cells were retained according to the following quality control criteria: nFeature_RNA > 200, nCount_RNA > 1000, and mitochondrial gene content < 20%. After quality filtering, 19,192 cells were included for subsequent analyses. Highly variable genes were identified using the “Find Variable Features” function, followed by principal component analysis (PCA) for dimensionality reduction. Cell type annotation was conducted based on established colon-specific marker genes reported in previous studies. Based on RUNX1 expression levels in tumor cells, the six CRC samples were stratified into RUNX1-high and RUNX1-low groups according to the median RUNX1 expression calculated across tumor cells within each sample.

### Bulk transcriptome data processing and analysis

Bulk RNA sequencing (RNA-seq) data of CRC were obtained from TCGA. Gene expression profiles were normalized using the Fragments Per Kilobase of Transcript per Million mapped reads (FPKM) method. After removing duplicated entries and samples with incomplete clinical or expression information, a total of 476 CRC samples from the TCGA-COAD cohort were included for downstream analyses. For stratified analyses, patients were divided into RUNX1-high and RUNX1-low groups based on the median expression level of RUNX1 across the TCGA-COAD cohort. Correlation analyses between RUNX1-high and GITRL or Foxp3 were performed using the Spearman rank correlation test implemented in the “dplyr” package (PMID: 34028547). Immune cell infiltration levels were estimated using the CIBERSORT algorithm implemented in the “cibersort” package (PMID: 31061481). Data visualization was conducted using the “ggplot2” package (Wickham, H. (2016). ggplot2: Elegant Graphics for Data Analysis. Springer, Cham).

### Statistical analysis

Data analysis was performed using SPSS software (version 22.0). The immunohistochemistry (IHC) scores of RUNX1, GITRL, and FOXP3 in 36 CRC tissue samples are expressed as the mean ± SEM. Animal experiments were replicated twice, with consistent trends observed between trials; the data shown represent the mean ± standard deviation (SD) from one independent experiment. Cytological experiments were repeated at least two to three times to ensure reproducibility; results are presented as the mean ± SD of one representative experiment. Comparisons between two groups were made using the Student’s t-test. A two-tailed *P*-value < 0.05 was considered statistically significant.

Regarding sample-size justification, our experimental design aligns with established norms in the field. For tissue-based analyses, we referenced the study “CCL2-CCR2 axis recruits tumor-associated macrophages to induce immune evasion through PD-1 signaling in esophageal carcinogenesis” (Molecular Cancer), in which IHC assessments were conducted on cohorts of 10, 22, and 58 tissue samples. For in vivo experiments, our group sizes of 4–5 mice are consistent with common practice in the field, as reflected in numerous published studies. We believe our sample sizes are appropriate for generating reliable and interpretable data within this research context.

## Supplementary information

Supplementary materials

uncropped gel

Original data of RNA-seq

Original data of Cut-Tag

Original data of Cut-Tag

Original data of Cut-Tag

## Data Availability

All data supporting the findings of this study are available from the corresponding author upon reasonable request.
