# Supplementary material for: RUNX1 restrains STAT1-GITRL signaling to shape an immunosuppressive CRC microenvironment
Source: Cell Death Discov. 2026 Mar 25;12:151. doi: 10.1038/s41420-026-03053-7 (PMC13040063; doi:10.1038/s41420-026-03053-7)
Supplement: Supplementary file 1 — Supplementary materials [file 41420_2026_3053_MOESM1_ESM.docx]

**Supplemental Figure 1**

**
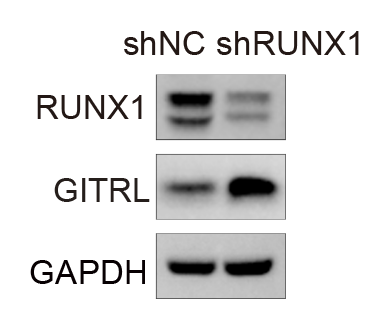
**

Supplemental Figure 1. Western blot analysis of RUNX1 and GITRL protein expression in SW480 cells following RUNX1 knockdown.

**Supplemental Figure 2**

**
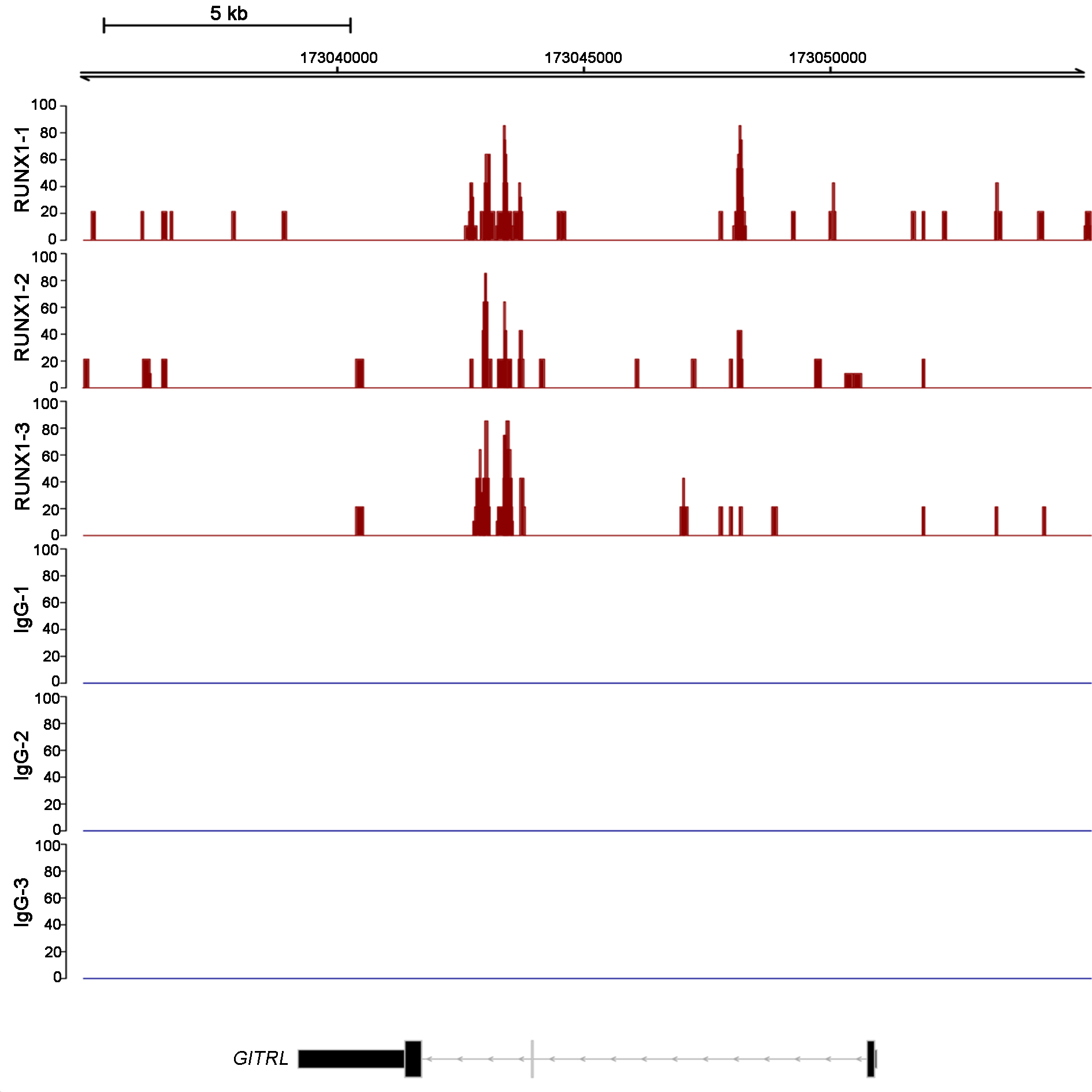
**

Supplemental Figure 2. The IGV visualization, including the corresponding peak track for GITRL with depth/scale and replicate information, demonstrates the absence of RUNX1 read enrichment within the GITRL promoter region.

**Supplemental Figure 3**

**
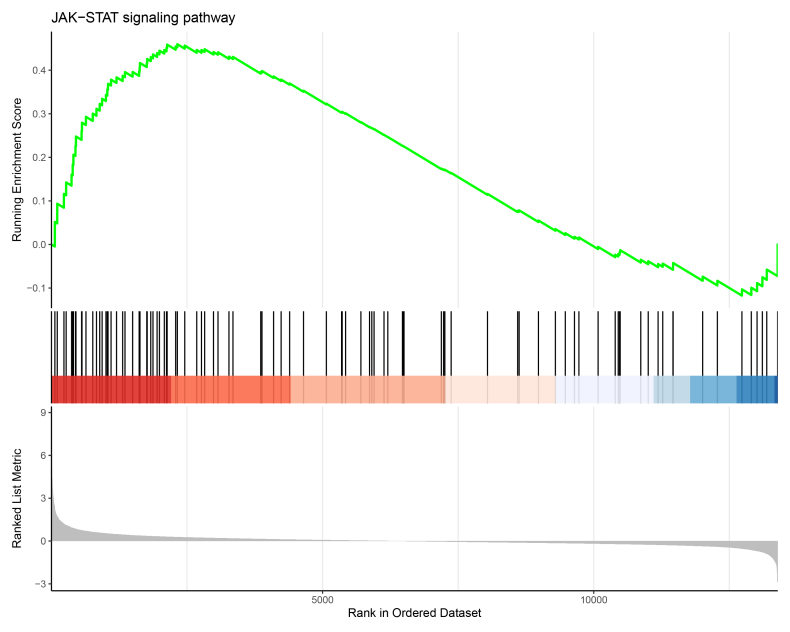
**

Supplemental Figure 3. The JAK-STAT signaling pathway was identified as significantly enriched through RNA sequencing and Gene Set Enrichment Analysis (GSEA).

**Supplemental Figure 4**


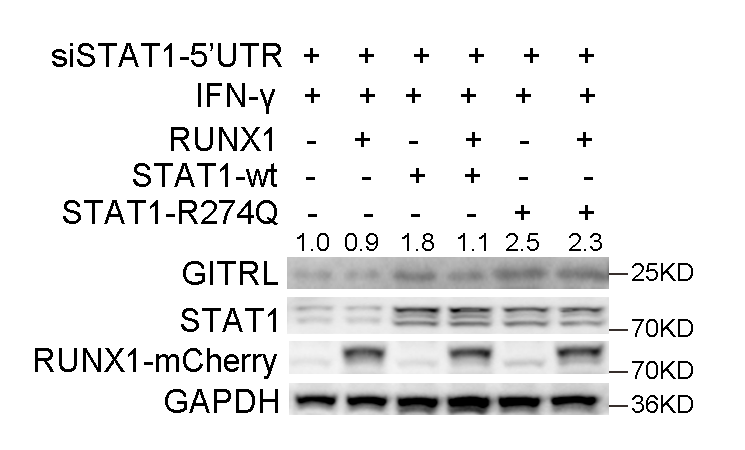


Supplemental Figure 4. Western blot analysis of GITRL expression in HCT116 cells with STAT1 knockdown. Cells were transfected with plasmids expressing RUNX1 (pcDNA3.1(+)-hRUNX1(V2)-3×FLAG-linker‑mCherry), wild‑type STAT1 (pcDNA3.1‑STAT1(wildtype)-3×Myc), or STAT1‑R274Q (pcDNA3.1‑STAT1(R274Q)-3×HA), followed by treatment with 100 U mL⁻¹ IFN‑γ for 48 h.

**Supplemental Figure 5**

**
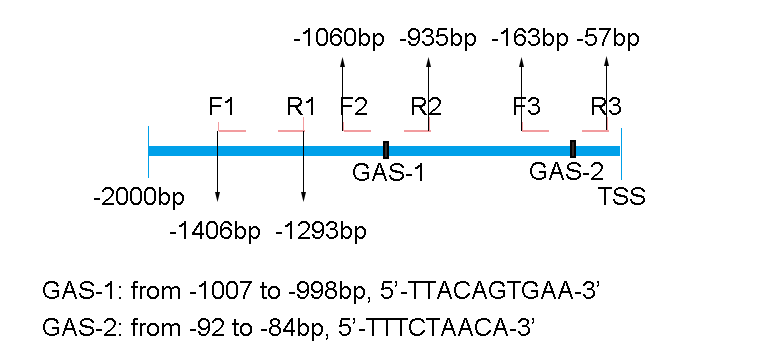
**

Supplemental Figure 5. The schematic diagram illustrates the primer locations for ChIP-qPCR and the potential GAS motifs within the GITRL promoter. The core human GAS motif is defined as 5’-TTNCNNNAA-3’. We identified two sequences within the PCR-amplified regions that match this pattern, which may represent functional GAS sites in the GITRL promoter.

**Supplemental Figure 6**

**
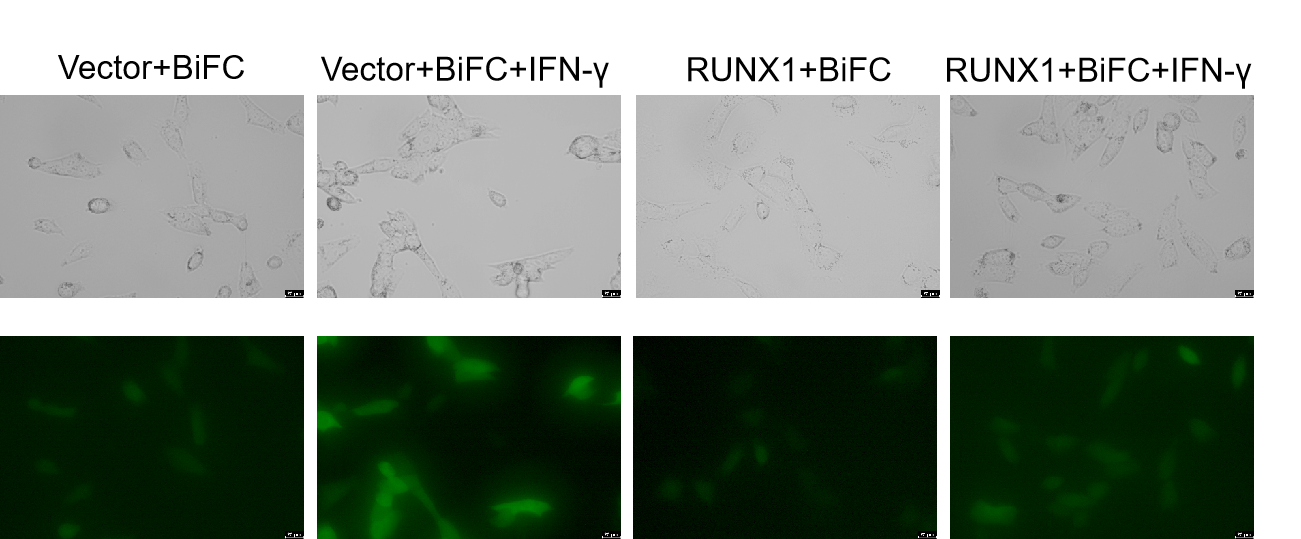
**

Supplemental Figure 6. A bimolecular fluorescence complementation (BiFC) assay was performed to visualize STAT1 dimerization in the presence or absence of IFN‑γ (100 U ml⁻¹, 48 h). STAT1 was fused either to the N‑terminal fragment of Cerulean (CN, residues 1‑172) or to the C‑terminal fragment of a cyan fluorescent protein (CC, residues 155‑238). Dimerization of STAT1 brings CN and CC into proximity, allowing reconstitution of a functional cyan fluorescent protein. Representative fluorescence images reflect the extent of complementation, which correlates with STAT1 dimerization.

**Supplemental Figure 7**


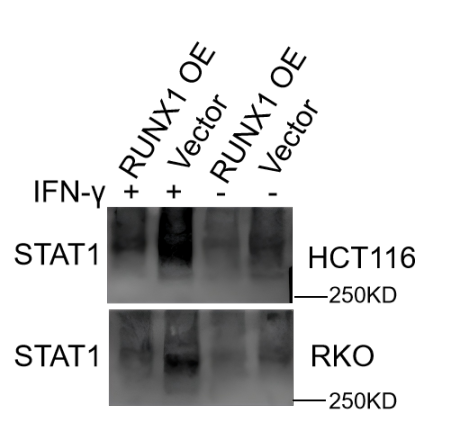


Supplemental Figure 7. Native-PAGE and Western blot analysis of STAT1 dimer formation in HCT116 and RKO cells overexpressing RUNX1, with or without treatment with 100 U ml⁻¹ IFN-γ for 48 h.

**Supplemental Figure 8**


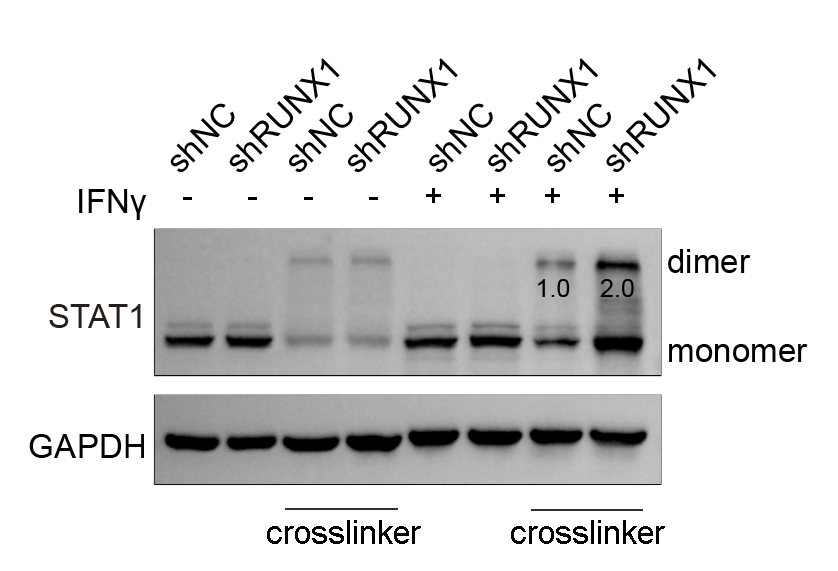


Supplemental Figure 8. Western blot analysis of STAT1 dimer and monomer expression in SW480 cells following RUNX1 knockdown, treated with or without 100 U·ml⁻¹ IFN-γ for 6 hours.

**Supplemental Figure 9**

**
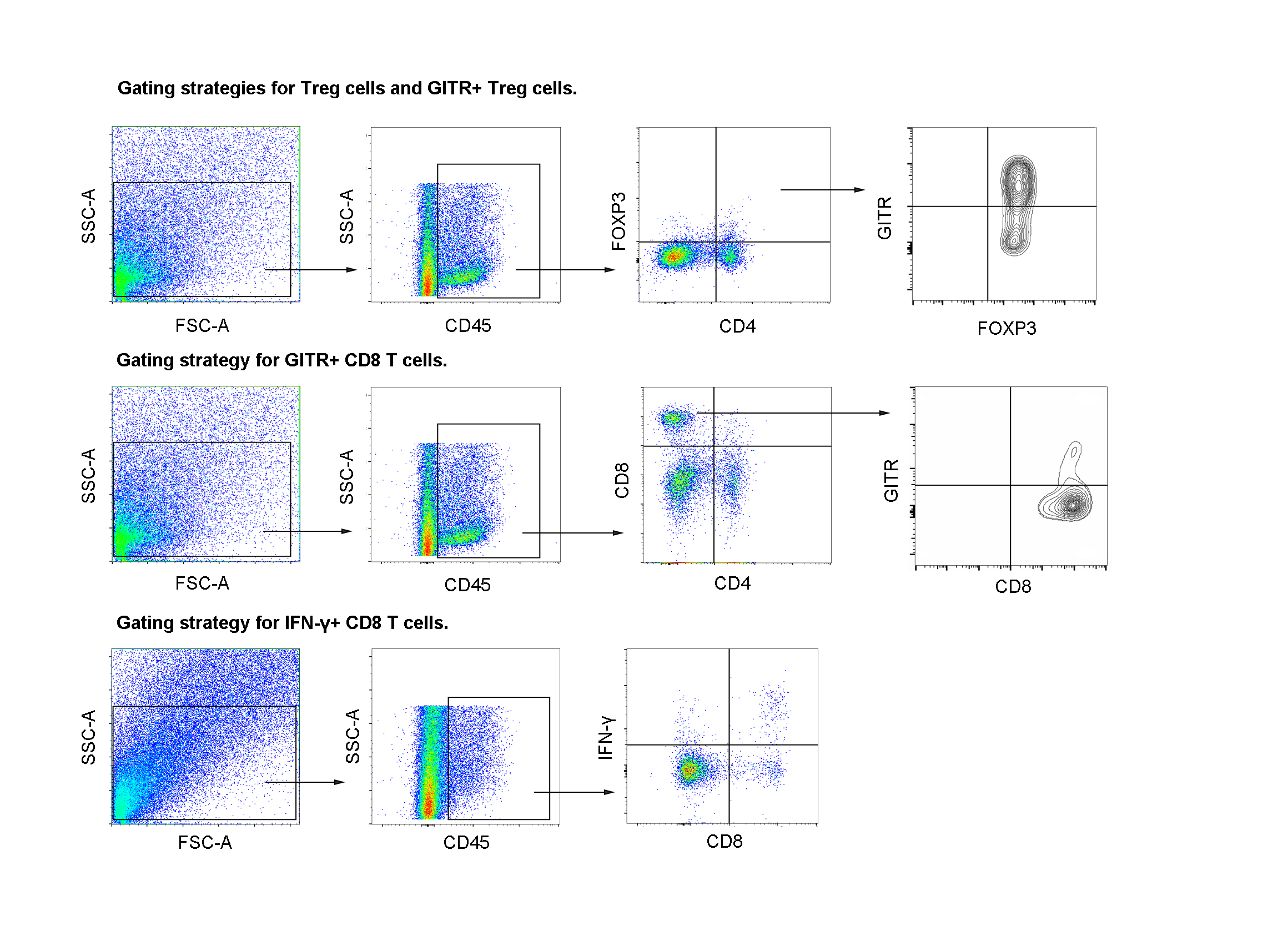
**

Supplemental Figure 9. The gating strategies of flow cytometry.

**Supplementary Table 1. The QC metrics of CUT&Tag.**

|  | RUNX1OE-1 | RUNX1OE-2 | RUNX1OE-3 | IgG-1 | IgG-2 | IgG-3 |
| --- | --- | --- | --- | --- | --- | --- |
| All reads | 15818203 | 15820182 | 15818564 | 443864 | 444445 | 444153 |
| Mapping reads | 15550068 | 15549551 | 15548713 | 383409 | 383361 | 383502 |
| Peak numbers | 38423 | 38393 | 38582 |  |  |  |
| FRIP | 36.10% | 36.05% | 36.10% |  |  |  |

**Supplementary Table 2. sh/siRNAs used for knockdown of targeted genes**

| **shRNAs** | **Sequence** |
| --- | --- |
| shRNA for hRUNX1 | 5’-GAACCACTCCACTGCCTTTAA-3’ |
| shRNA for hSTAT1 | 5’-CGGTTGAACCCTACACGAA-3’ |
| shRNA for mGITRL | 5’-GCACATGACATCTCCCAAACC-3’ |
| siRNA for hSTAT1 | 5’-CGGUUGAACCCUACACGAATT-3’ |
| siRNA for hSTAT1-5’UTR | 5’-CAGCUGAACUUGUUGAGAUTT-3’ |

**Supplementary Table 3. qPCR primers**

| **Primers** | **Sequence** |
| --- | --- |
| RUNX1-F | CTGCCCATCGCTTTCAAGGT |
| RUNX1-R | GCCGAGTAGTTTTCATCATTGCC |
| GAPDH-F | TGACTTCAACAGCGACACCC |
| GAPDH-R | CTGGTGGTCCAGGGGTCTTA |
| GITRL-F | AGTGGCTCCCAATGCAAACTA |
| GITRL-R | TATACAGCCGCACCTCAAAAG |
| STAT1-F | CAGCTTGACTCAAAATTCCTGGA |
| STAT1-R | TGAAGATTACGCTTGCTTTTCCT |
| HES1-F | TCAACACGACACCGGATAAAC |
| HES1-R | GCCGCGAGCTATCTTTCTTCA |
| NR1D1-F | TGGACTCCAACAACAACACAG |
| NR1D1-R | GATGGTGGGAAGTAGGTGGG |
| CDKN1C-F | GCGGCGATCAAGAAGCTGT |
| CDKN1C-R | GCTTGGCGAAGAAATCGGAGA |
| TRIM6-F | ACTGGGTTGACGTGACCCT |
| TRIM6-R | TCCCACAAACCTCACTTGTCT |
| IKBKE-F | TGCCTGAGGATGAGTTCCTG |
| IKBKE-R | CGATGCACAATGCCGTTCT |
| RGCC-F | CGCCACTTCCACTACGAGG |
| RGCC-R | CAGCAATGAAGGCTTCTAGCTC |
| DDIT3-F | GGAAACAGAGTGGTCATTCCC |
| DDIT3-R | CTGCTTGAGCCGTTCATTCTC |
| STING1-F | GGCCCGGATTCGAACTTACA |
| STING1-R | GTCATCTGCAGGTTCCGCT |
| PER1-F | GCCAACCAGGAATACTACCAGC |
| PER1-R | GTGTGTACTCAGACGTGATGTG |
| RFPL2-F | ACAAAATCAGGCGCAATCGG |
| RFPL2-R | AGGATGCAAACGGACACGTC |
| AFAP1L2-F | CTCCTCCGGCTTTACACCAAA |
| AFAP1L2-R | GGCAAGCTGTTTCCGTTCTG |
| ARRDC4-F | TCTCGGAGGTGGAGTACCTG |
| ARRDC4-R | CCCAGTAAACGAGGTGACCAAA |
| BIRC3-F | AAGCTACCTCTCAGCCTACTTT |
| BIRC3-R | CCACTGTTTTCTGTACCCGGA |
| TGFBR3-F | TGGGGTCTCCAGACTGTTTTT |
| TGFBR3-R | CTGCTCCATACTCTTTTCGGG |
| CSF1R-F | GGGAATCCCAGTGATAGAGCC |
| CSF1R-R | TTGGAAGGTAGCGTTGTTGGT |
| TSC22D1-F | AGCGTCAGGTCCCGTTTTC |
| TSC22D1-R | CTGGTGCATTGTGTTGGGT |
| WLS-F | TCCCTGGCTTACCGTGATGA |
| WLS-R | GCATTTGAGTTTCCGTGGTACT |
| SOX4-F | AGCGACAAGATCCCTTTCATTC |
| SOX4-R | CGTTGCCGGACTTCACCTT |
| CXCL1-F | AGGCAGGGGAATGTATGTGC |
| CXCL1-R | AGCCCCTTTGTTCTAAGCCA |
| MMP28-F | TCCCACCTCCACTCGATTCAG |
| MMP28-R | GCCGCATAACTGTTGGTATCT |
| STK10-F | ATCCTGCGCCTGTCTACCTT |
| STK10-R | GCCTTGTAAACCTTGCCGAA |
| SERPINE1-F | ACCGCAACGTGGTTTTCTCA |
| SERPINE1-R | TTGAATCCCATAGCTGCTTGAAT |
| PTK2B-F | CCCCTGAGTCGAGTAAAGTTGG |
| PTK2B-R | GATACGCACGTCCTCCTTTTC |
| EDN1-F | AGAGTGTGTCTACTTCTGCCA |
| EDN1-R | CTTCCAAGTCCATACGGAACAA |
| LCK-F | TGCCATTATCCCATAGTCCCA |
| LCK-R | GAGCCTTCGTAGGTAACCAGT |
| MMP14-F | GGCTACAGCAATATGGCTACC |
| MMP14-R | GATGGCCGCTGAGAGTGAC |
| GAS6-F | GGTAGCTGAGTTTGACTTCCG |
| GAS6-R | GACAGCATCCCTGTTGACCTT |
| DDT-F | GGAGCTGGACACGAATTTGC |
| DDT-R | ACTACGCCGATGGAGGAGAT |
| PDGFB-F | CTCGATCCGCTCCTTTGATGA |
| PDGFB-R | CGTTGGTGCGGTCTATGAG |
| LGALS9-F | TCTGGGACTATTCAAGGAGGTC |
| LGALS9-R | CCACTGGAGCTGAGAACGG |
| CXCR1-F | CTGACCCAGAAGCGTCACTTG |
| CXCR1-R | CCAGGACCTCATAGCAAACTG |
| PTGER4-F | CCGGCGGTGATGTTCATCTT |
| PTGER4-R | CCCACATACCAGCGTGTAGAA |
| CXCL8-F | TCTGCAGCTCTGTGTGAAGG |
| CXCL8-R | TTCTCAGCCCTCTTCAAAAACT |
| SAA1-F | CAGGTGAGGAGCACACCAAG |
| SAA1-R | CCATCAAAAGCCTCGCCAAG |
| F3-F | GGCGCTTCAGGCACTACAA |
| F3-R | TTGATTGACGGGTTTGGGTTC |
| NRP1-F | GGCGCTTTTCGCAACGATAAA |
| NRP1-R | TCGCATTTTTCACTTGGGTGAT |
| THBS1-F | AGACTCCGCATCGCAAAGG |
| THBS1-R | TCACCACGTTGTTGTCAAGGG |

**Supplementary Table 4. ChIP-qPCR primers**

| **Primers** | **Sequence** |
| --- | --- |
| Forward primer for site1 | TGGGCTTGAGTTTTCACTCC |
| Reverse primer for site1 | GTCCAGAGATTTAACATCCTCCTT |
| Forward primer for site2 | CACCTTCATTTCATTCTCCACA |
| Reverse primer for site2 | CCCAGTTCCTACCTGCTCTG |
| Forward primer for site3 | AGCCTTCCGTTCCTTTTGTT |
| Reverse primer for site3 | GAAGGCGGAGTTTTCCTTCT |

**Supplementary Table 5. The antibody information.**

| **Antibody** | **Clone** | **Source** | **Dilution** | **Brand** | **Cat No** |
| --- | --- | --- | --- | --- | --- |
| GITRL | Polyclonal | Rabbit | 1:1000 | Novus | NBP1-77240 |
| RUNX1 | Polyclonal | Rabbit | 1:1000 | Proteintech | 25315-1-AP |
| STAT1 | Polyclonal | Rabbit | 1:1000 | Proteintech | 10144-2-AP |
| phospho-STAT1 | Polyclonal | Rabbit | 1:1000 | Proteintech | 28979-1-AP |
| HA tag | Polyclonal | Rabbit | 1:1000 | Proteintech | 51064-2-AP |
| Myc tag | 1A5A2 | Mouse | 1:1000 | Proteintech | 60003-2-Ig |
| Lamin B1 | Polyclonal | Rabbit | 1:1000 | Proteintech | 12987-1-AP |
| GAPDH | 1E6D9 | Mouse | 1:1000 | Proteintech | 60004-1-Ig |
| CD45 | 30-F11 | Rat | 1:200 | eBioscience | 56-0451-80 |
| CD4 | GK1.5 | Rat | 1:200 | eBioscience | 48-0041-80 |
| CD8 | eBioH35-17.2 | Rat | 1:200 | eBioscience | 17-0083-81 |
| GITR | DTA-1 | Rat | 1:200 | eBioscience | 12-5874-82 |
| IFN-γ | XMG1.2 | Rat | 1:100 | eBioscience | 12-7311-82 |
| FOXP3 | FJK-16s | Rat | 1:100 | eBioscience | 45-5773-80 |
| FOXP3 | EPR15038-69 | Rabbit | 1:100 | Abcam | Ab191416 |
| GITRL | Polyclonal | Rabbit | 1:1500 | Proteintech | 23899-1-AP |
| Flag tag | M2 | Mouse | 1:50 | Sigma | F1804 |
| STAT1 | Polyclonal | Rabbit | 1:50 | CST | 9172 |
| FOXP3 | UMAB248 | Mouse | 1:1500 | Origene | UM870140 |
| STAT1 | G3-B11 | Mouse | 1:100 | HUABIO | M1407-1 |

**Supplementary Information.**

**BiFC assay**

HCT116 cells were seeded in 6-well plates and transfected with plasmids encoding STAT1-CN or STAT1-CC, either in the presence or absence of a RUNX1 overexpression construct. Following transfection, the cells were treated with IFN‑γ (100 U·mL⁻¹) and incubated for 48 h to allow for protein expression. BiFC signals were visualized using fluorescence microscopy (LEICA DMi8). All plasmid constructs were generated with the technical support of HanYi Biosciences (Guangzhou, China).

**Native-PAGE analysis**

HCT116 and RKO cells were seeded into 6-well plates and transfected with either a RUNX1-encoding plasmid or an empty vector control. Following transfection, cells were cultured for 48 h to permit protein expression. IFN-γ (100 U ml⁻¹) was added after the transfection step. STAT1 dimer formation was then analyzed under non-denaturing conditions using native PAGE (P0016N, Solarbio) followed by Western blotting.

**Abbreviations**

| RUNX1 | runt-related transcription factor 1 |
| --- | --- |
| TME | tumor immune microenvironment |
| CRC | colorectal cancer |
| GITRL | glucocorticoid-induced tumor-necrosis-factor-receptor-related protein ligand |
| GITR | glucocorticoid-induced tumor-necrosis-factor-receptor-related protein |
| PD-1 | programmed death receptor 1 |
| IHC | immunohistochemical |
| TNFRSF | tumor necrosis factor receptor superfamily |
| Ab | antibody |
| Treg | regulatory T cell |
| STAT1 | signal transducer and activator of transcription 1 |
| ICB | immune checkpoint blockade |
| CoR | co-stimulatory receptor |
| FDA | food and drug administration |
| MSI-H | high levels of microsatellite instability |
| MSS | microsatellite stable |
| MSI-L | MSI-low |
| CTL | cytotoxic T lymphocyte |
| TCR | T-cell receptor |
| APC | antigen-presenting cell |
| RNA-seq | RNA sequencing |
| CUT&Tag | high-throughput cleavage under targets & tagmentation |
| LRA | luciferase reporter assay |
| ChIP | chromatin immunoprecipitation |
| qPCR | quantitative polymerase chain reaction |
| IFN-γ | interferon-γ |
| EMT | epithelial-mesenchymal transition |
| EGCG | epigallocatechin gallate |
| ATCC | american type culture collection |
| IRS | immuno-reactivity scores |
| qRT-PCR | quantitative real-time PCR |
| CO-IP | co-immunoprecipitation |
| MS | mass spectrometry |
| shNC | shRNA for negative control |
| shRUNX1 | shRNA for RUNX1 |
| shGITRL | shRNA for GITRL |
| OE | overexpression |
| s.c | subcutaneously |
| i.p | intraperitoneally |
| Kb | kilobases |
| bp | base pair |
| chr | chromosome |
| TSS | transcription start site |
| IP | immunoprecipitation |
| ns | not statistically |
| Luc | luciferase |
| F | forward primer |
| R | reverse primer |
| siSTAT1 | siRNA for STAT1 |
| PMA | phorbol myristate acetate |
| BFA | brefeldin A |
| GAS | gamma-activated sequence |
| PD-1 | programmed cell death protein 1 |
| DR3 | death receptor 3 |
| HVEM | herpesvirus entry mediator |
| TNFR3 | tumor necrosis factor receptor 3 |
| TCGA | the cancer genome atlas |
| DFS | disease free survival |
| TGFBR3 | transforming growth factor beta receptor 3 |
| CXCL8 | C-X-C Motif Chemokine Ligand 8 |
| FBS | fetal bovine serum |
| SD | standard deviation |
| shRNA | short hairpin RNA |
| scRNA-seq | single-cell RNA sequencing |
| PCA | principal component analysis |
| FPKM | fragments per kilobase of transcript per million mapped reads |
