# Supplementary material for: RUNX1 restrains STAT1-GITRL signaling to shape an immunosuppressive CRC microenvironment
Source: Cell Death Discov. 2026 Mar 25;12:151. doi: 10.1038/s41420-026-03053-7 (PMC13040063; doi:10.1038/s41420-026-03053-7)

Full unedited gel for Figure 1F

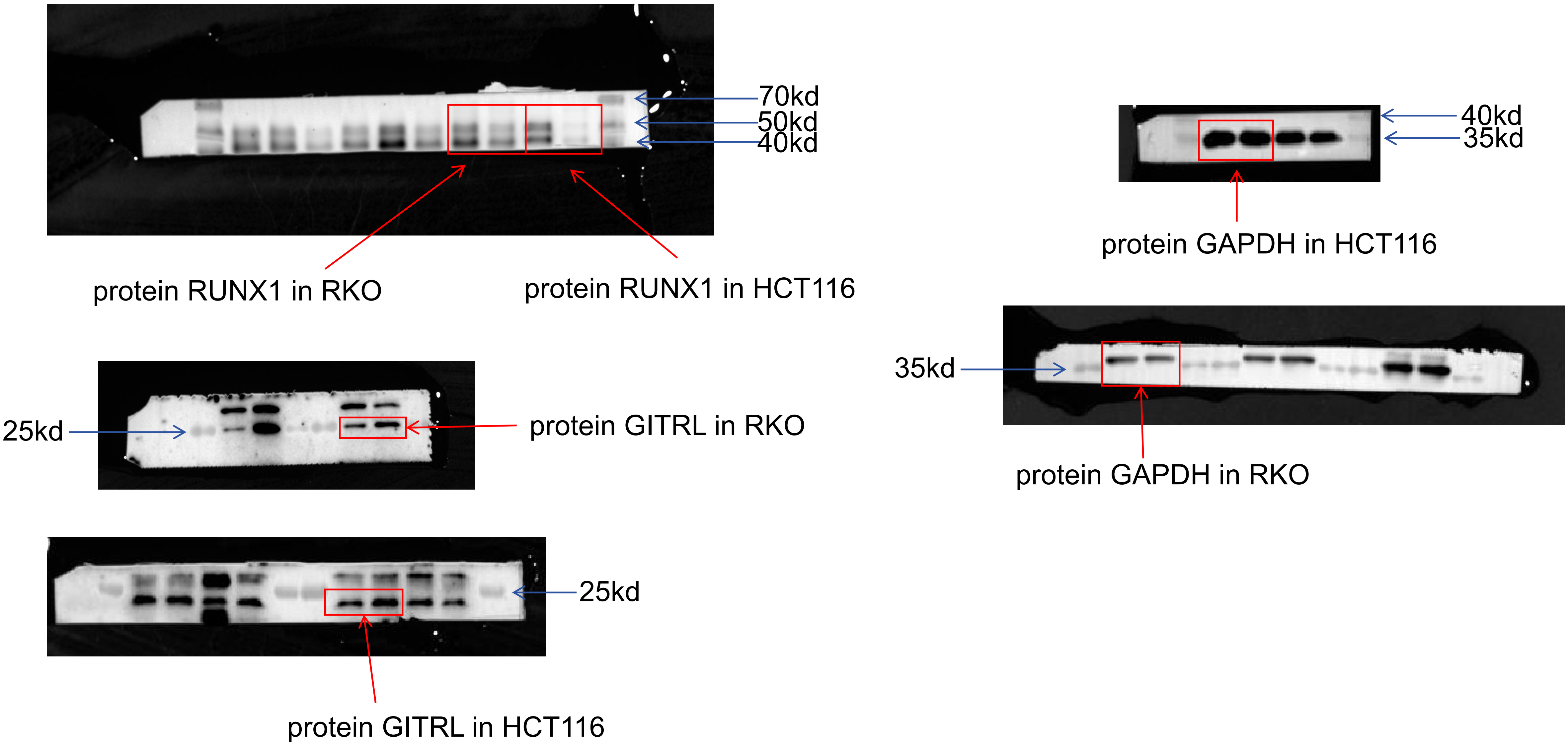

Full unedited gel for Figure 2A

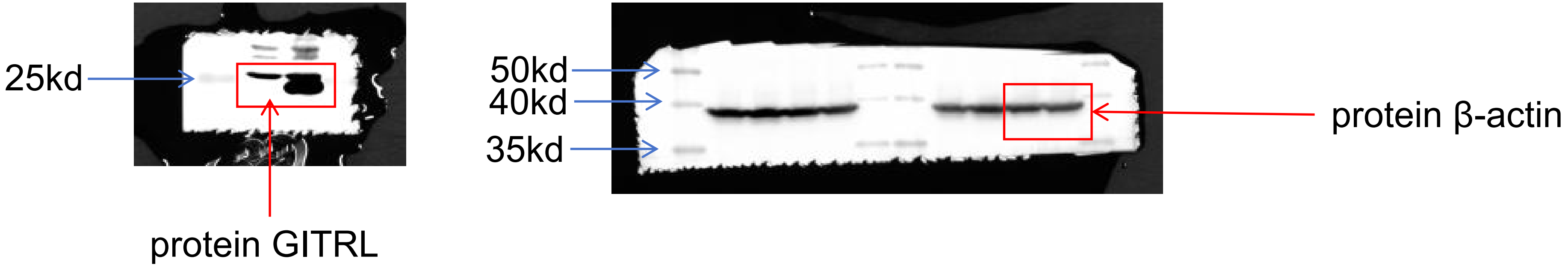

Full unedited gel for Figure 3A

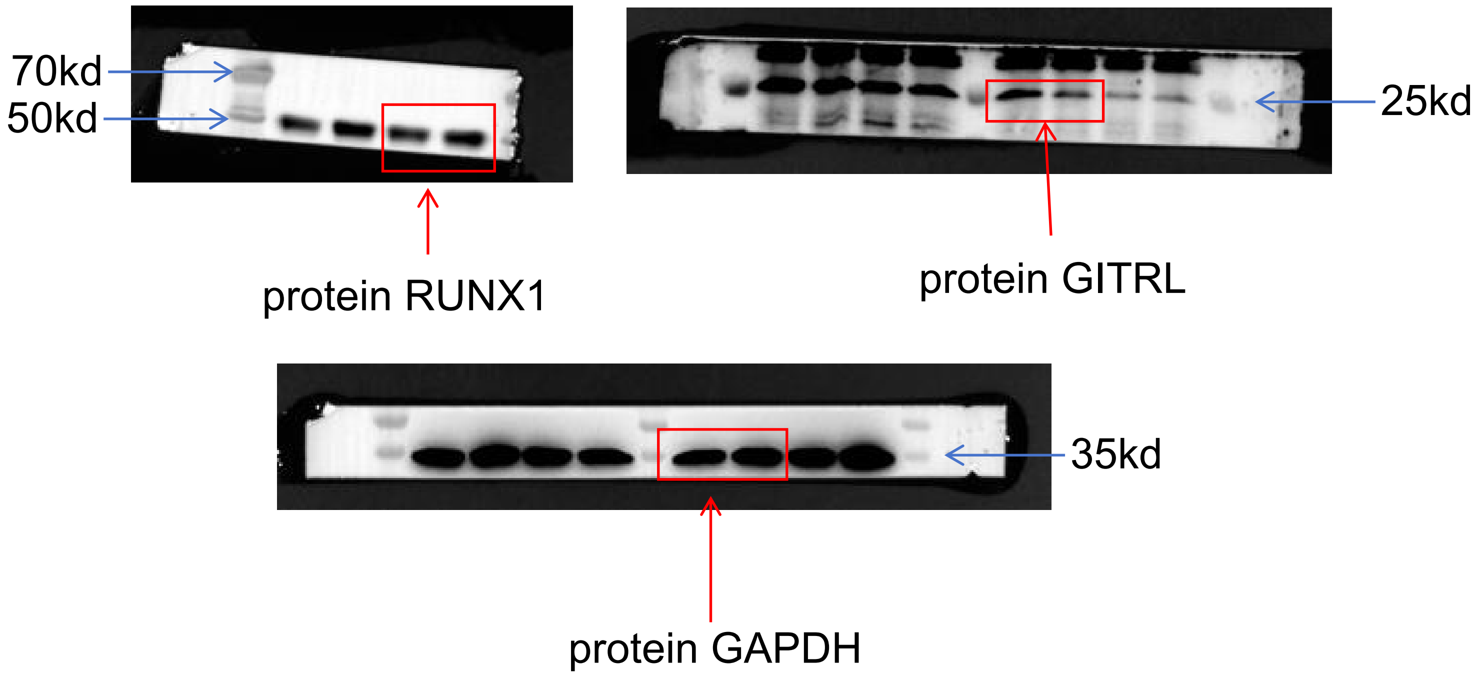

Full unedited gel for Figure 3B

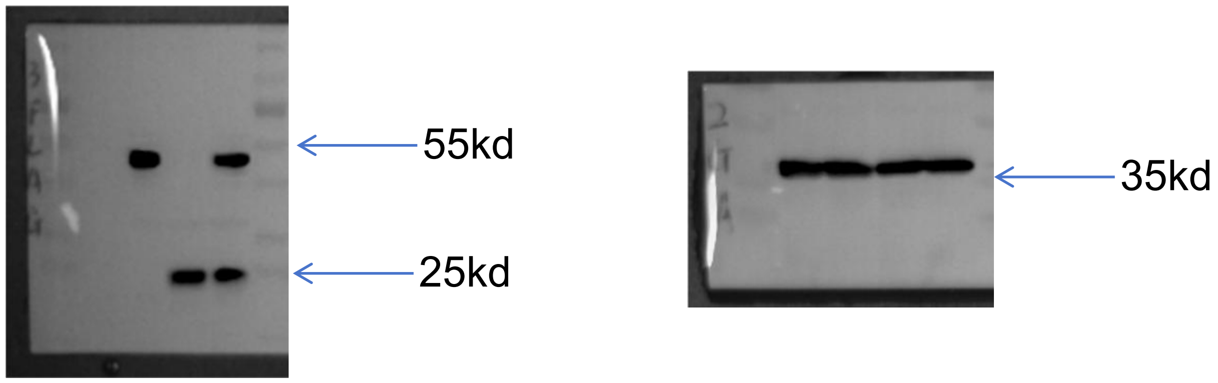

Full unedited gel for Figure 5D

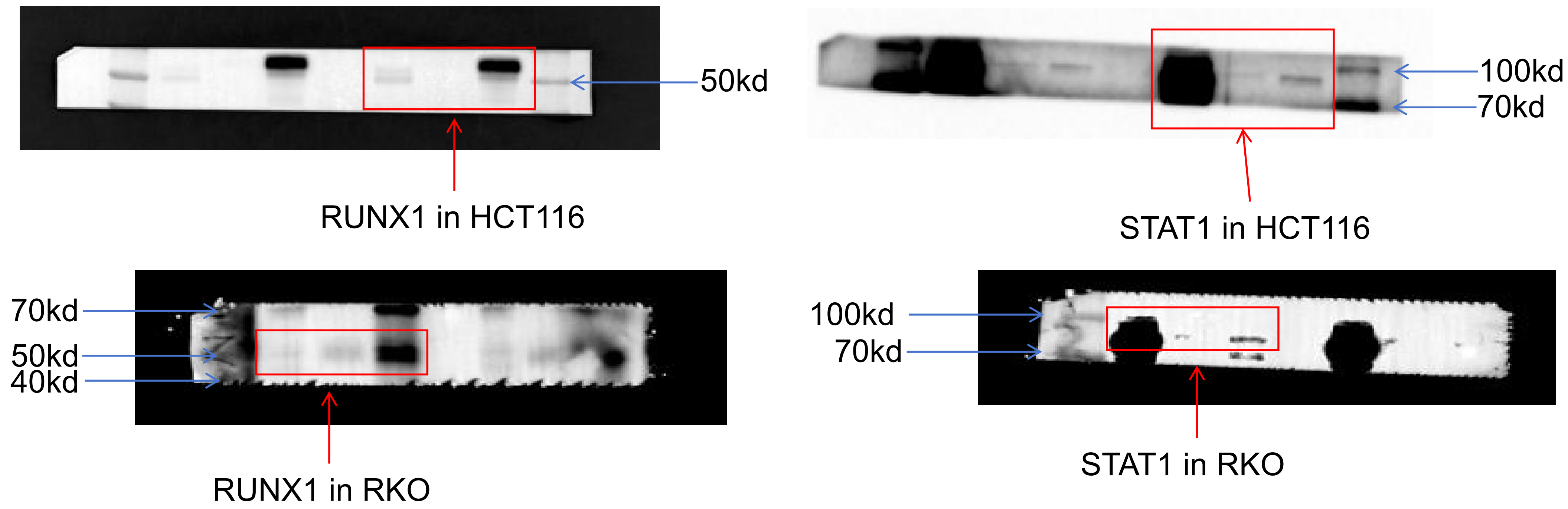

Full unedited gel for Figure 5E

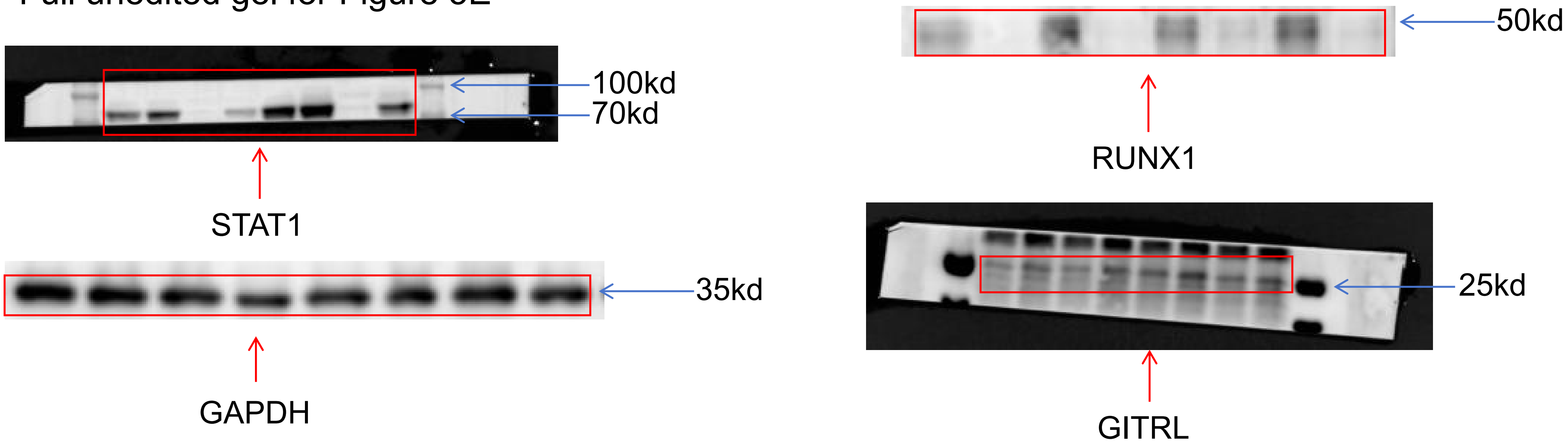

Full unedited gel for Figure 6A

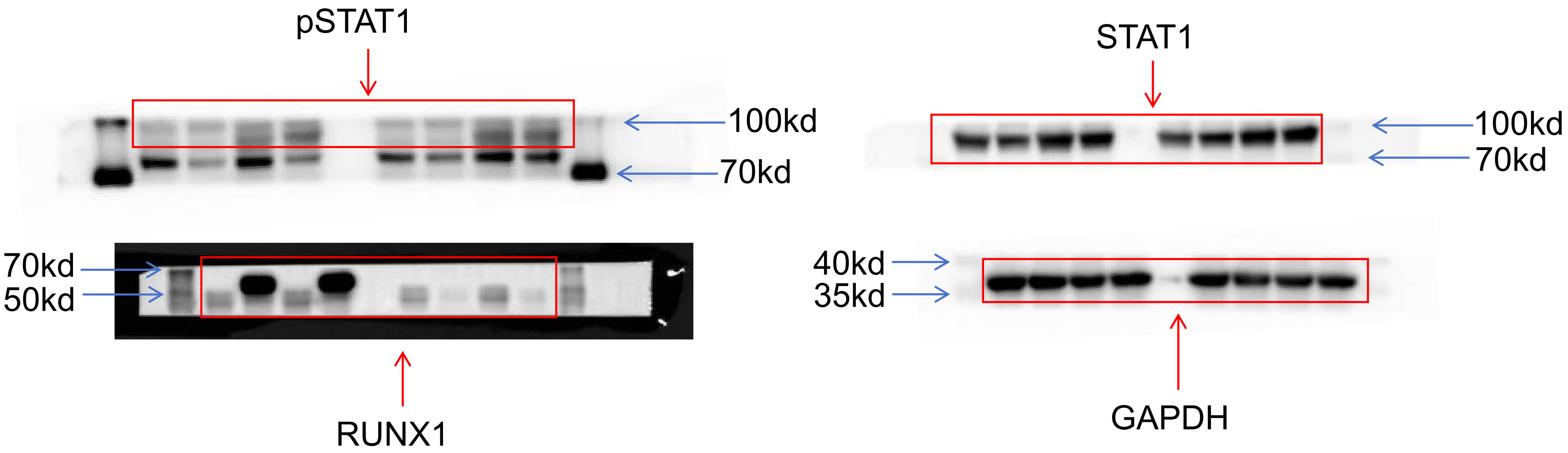

Full unedited gel for Figure 6B

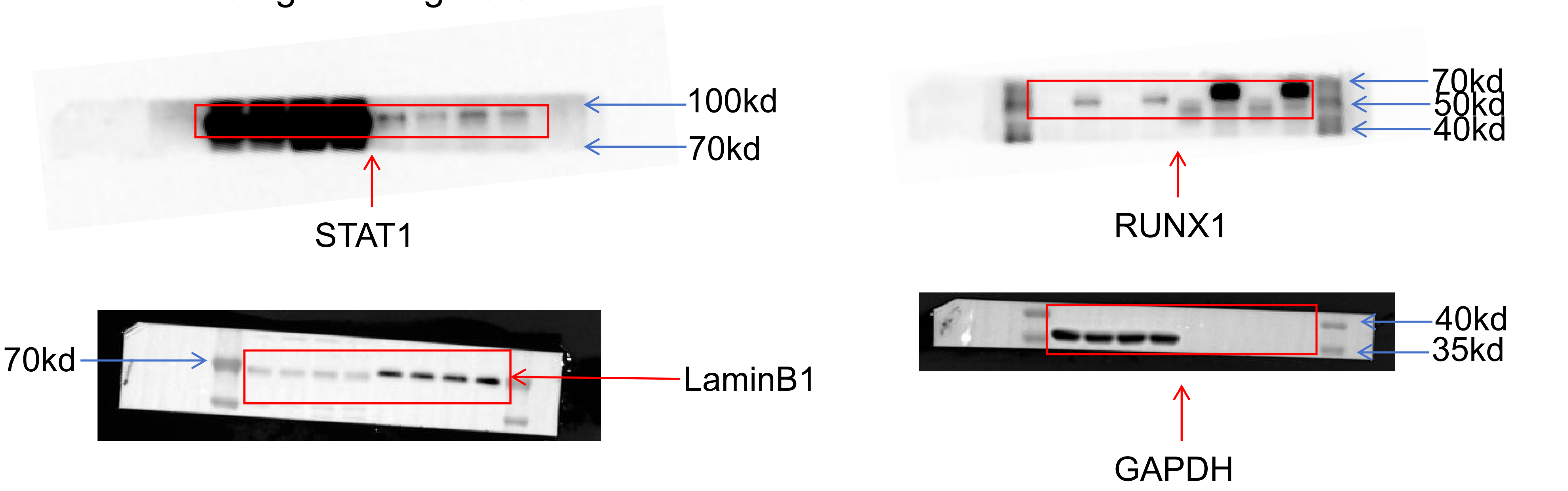

Full unedited gel for Figure 6C

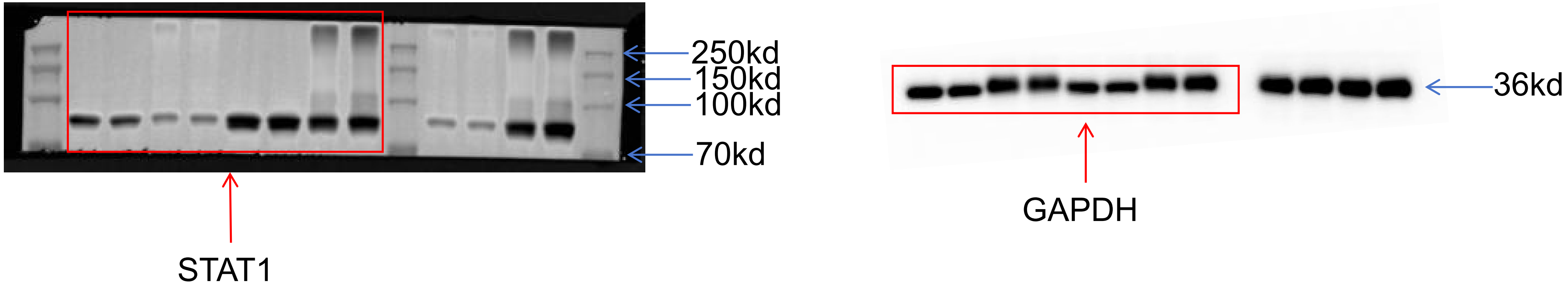

Full unedited gel for Figure 6D

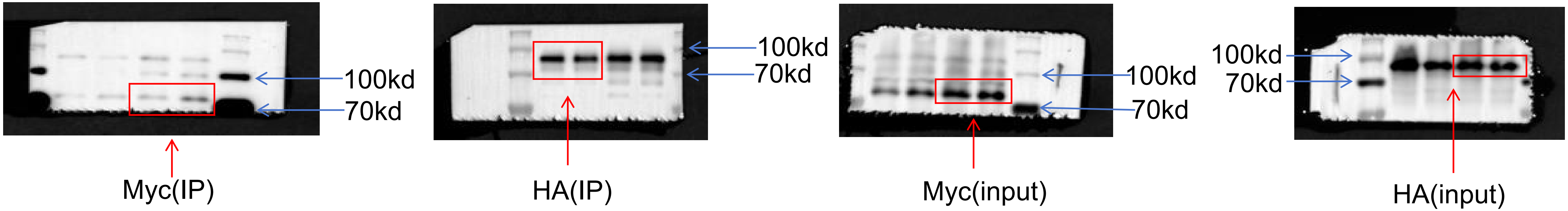

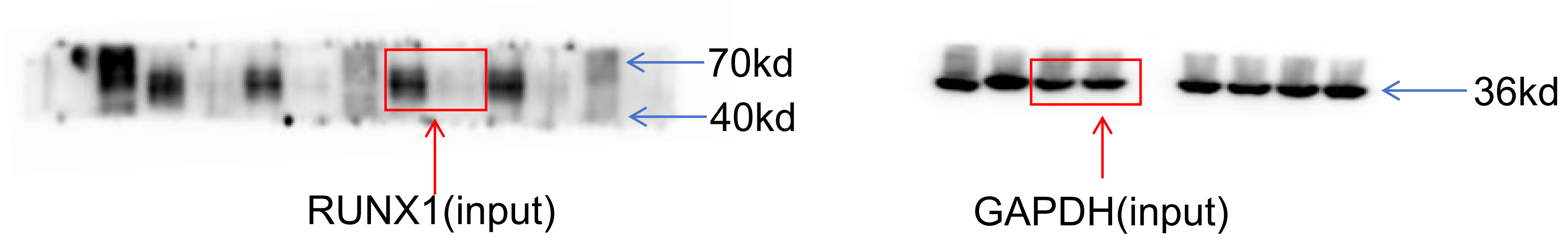

Full unedited gel for Figure 6F

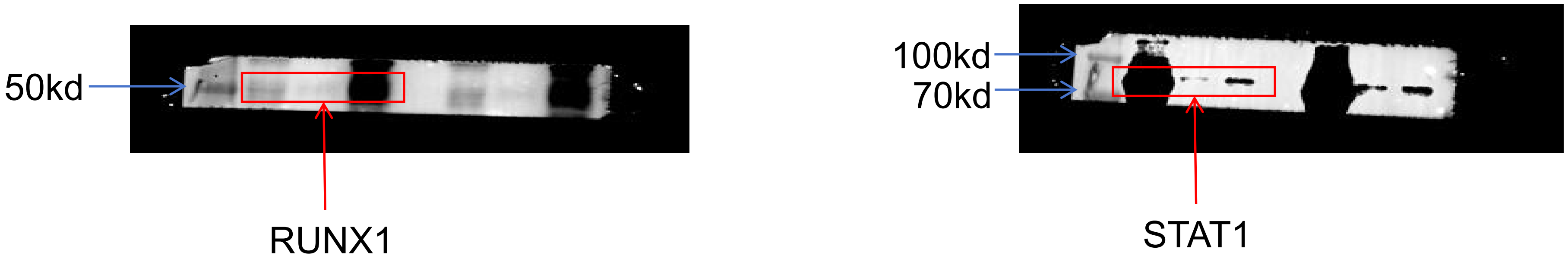

Full unedited gel for Figure 6G

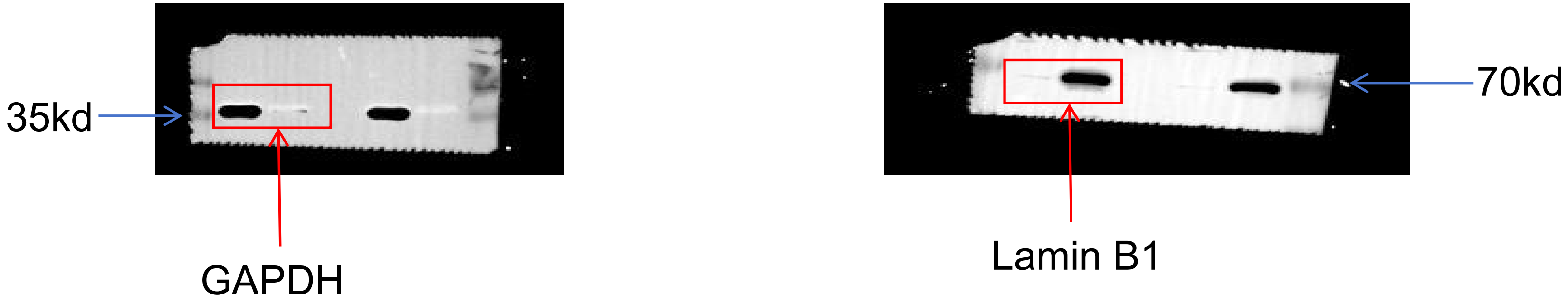

Full unedited gel for supplementary figure 1

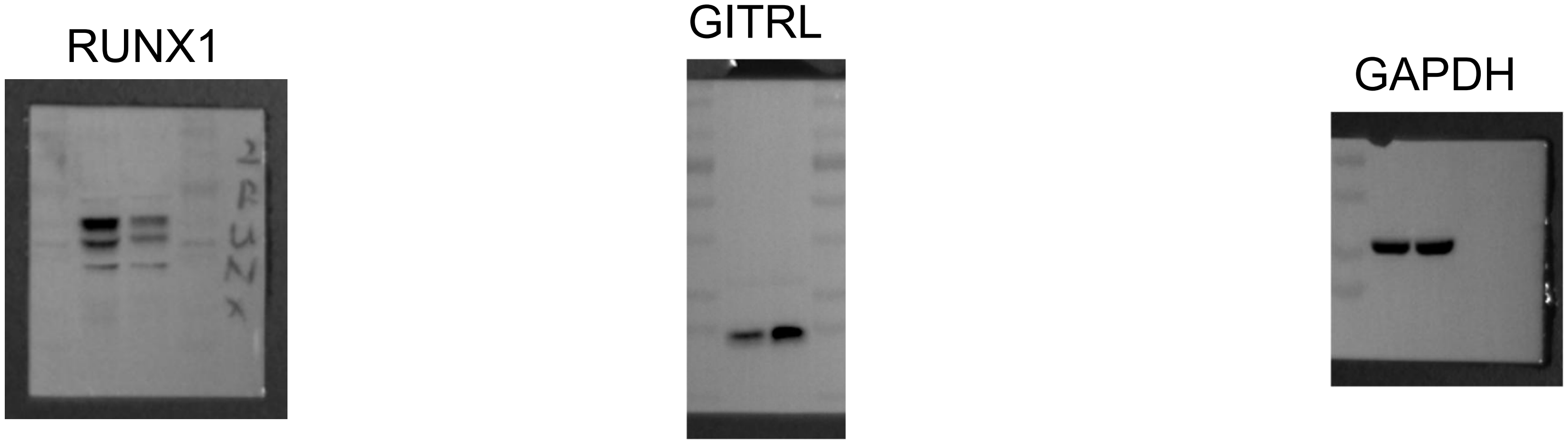

Full unedited gel for supplementary figure 1

RUNX1

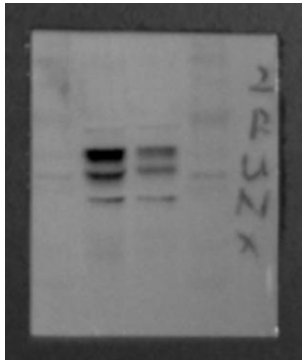

GITRL

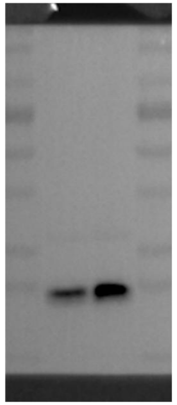

GAPDH

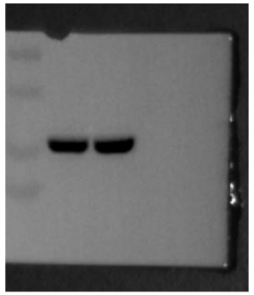

Full unedited gel for supplementary figure 8

STAT1

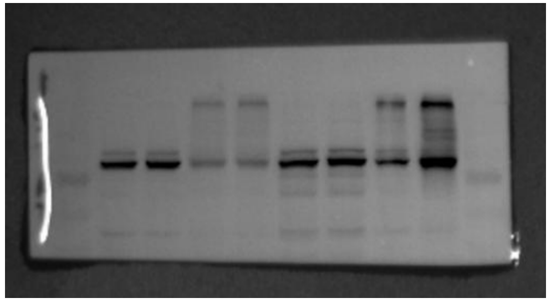

GAPDH

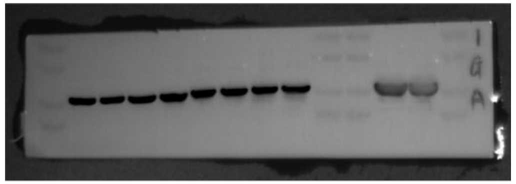

Supplement: Supplementary file 2 — uncropped gel [file 41420_2026_3053_MOESM2_ESM.pdf]
